# Verbal attacks on terrorist groups increase violence against civilians

**DOI:** 10.1093/pnasnexus/pgae437

**Published:** 2024-10-02

**Authors:** Iliyan Iliev, Nahrain Bet Younadam, Brandon J Kinne

**Affiliations:** School of Social Science and Global Studies, University of Southern Mississippi, Hattiesburg, MS 39406, USA; School of Government and Public Policy,University of Arizona, Tucson, AZ 85719, USA; Department of Political Science, University of California, Davis, CA 95616, USA

**Keywords:** Islamic State, terrorism, verbal conflict, bargaining, Bayesian vector autoregression

## Abstract

Terrorists and other transnational extremist groups are responsible for thousands of civilian deaths. In confronting extremists, governments have relied heavily on threats, demands, denunciations, and other forms of *verbal conflict.* Do these efforts at verbal coercion have any effect on terrorist behavior? This analysis focuses on the Islamic State in Iraq and Syria (ISIS), which continues to be the world's deadliest terrorist group and was responsible for recent high-profile attacks in Baghdad, Vienna, Kabul, and Russia. We use Bayesian structural vector autoregression models to analyze daily event data on interactions between ISIS and foreign governments for the 2014–2020 period. We find that verbal conflict initiated by governments not only failed to deter ISIS but in fact increased the frequency of ISIS's attacks on civilians. Additional empirical analyses, combined with evidence from ISIS's publications and public statements, suggest that this effect resulted from a perceived *credibility deficit*. Extremists use terror attacks to signal that they have the capabilities and willingness to inflict pain and suffering on civilian targets. Government attempts to coerce extremist groups verbally, rather than militarily, reflect an underestimation of the group's capabilities and resolve. In an effort to solidify their reputations, extremists engage in further violence toward civilians, thus leading to worse humanitarian consequences. We extend the analysis to Al-Qaeda in Iraq and Boko Haram and find similar results.

**Significance Statement**

Governments continue to struggle with how to best respond to terrorism and other forms of extremism. Leaders face domestic pressure to confront extremists, but military actions are risky. By turning instead to threats, denunciations, and other “verbal attacks,” a government can publicly register its disapproval with extremists while avoiding the costs of military confrontation. Due to their perceived low risk, such approaches continue to be widely used by governments. We find, however, that verbal attacks inflict unexpected costs. When governments targeted ISIS and similar extremist groups with verbal attacks, the organizations responded by attacking civilians. Though seemingly benign, verbal attacks have perverse humanitarian consequences that must be taken seriously when governments confront extremists.

## Introduction

On 2014 June 12, militants from the Islamic State in Iraq and Syria (ISIS) launched a surprise attack on Camp Speicher in Tikrit, Iraq, killing nearly 2,000 unarmed cadets in one of the deadliest terror attacks in modern history (1). In early August of that same year, ISIS initiated a genocidal campaign against Iraq's Yazidi population, ultimately leaving 5,000 Yazidis dead and thousands more captive (2). In early July 2016, ISIS militants coordinated a series of bombings in Baghdad that killed over 300 civilians (3). More recently, ISIS has been linked to attacks in Baghdad (4), Vienna (5), and Kabul (6). This systematic campaign of violence against civilians—characterized by mass executions, beheadings, crucifixions, armed assaults, bombings, and other extreme tactics—remains a global security concern not only due to the threat of further ISIS attacks, but also due to the potential for similar attacks by ISIS's inevitable successors. Although ISIS remains the world's deadliest terrorist organization (7), other well-known transnational extremist groups, such as Boko Haram and Al-Qaeda in Iraq (AQI), have relied on similar tactics (8, 9).

**Fig. 1.**
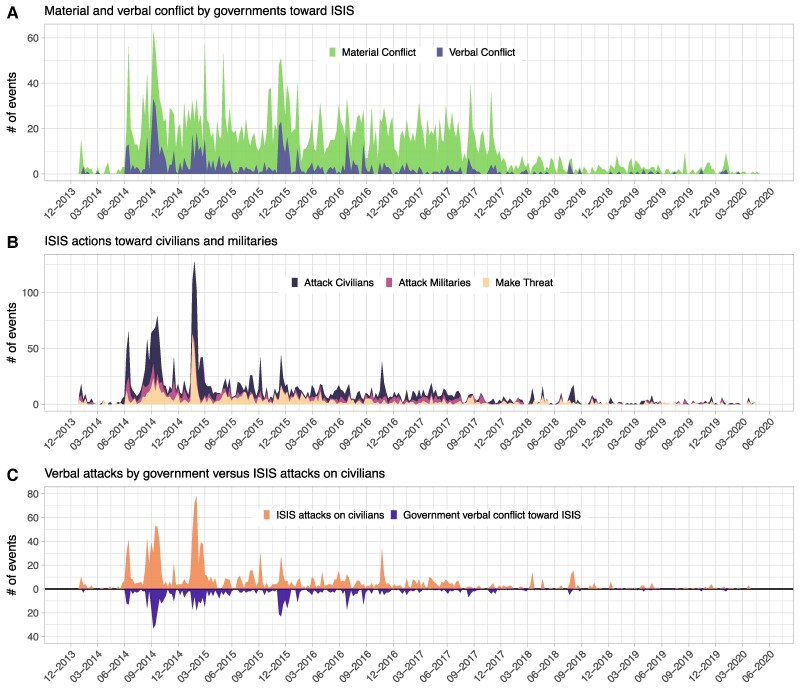
Daily event counts of government strategies and ISIS behaviors. All figures based on data from ICEWS (18). In (A), *material conflict* includes conventional uses of force by governments, military, and security forces against ISIS targets and *verbal conflict* includes investigations, demands, disapprovals, rejections, threats, and reductions in relations by governments toward ISIS. In (B), *attack civilians* includes acts of coercion, assault, and unconventional mass violence by ISIS against nonmilitary targets, including civilians, educational institutions and affiliates, journalists and news media, and businesses; *attack militaries* includes acts of coercion, assault, and unconventional violence by ISIS toward military targets; and *make threat* includes any verbal threat against any target. (C) Mirrored counts of *verbal conflict* events and *attack civilians* events. See SI Appendix A.3 for the specific CAMEO codes used to define each metric.

When responding to terrorists, governments have utilized two basic approaches. The first involves *material conflict:* airstrikes, ground campaigns, and other forms of direct military engagement. The second involves *verbal conflict:* making demands, rejecting extremists’ own demands, launching investigations, issuing formal disapprovals, and threatening to intervene or retaliate (10). While both approaches are widely evident in government confrontations with ISIS, verbal conflict was especially prevalent early in the crisis, when many governments faced political pressure to address the atrocities but were reluctant to confront ISIS militarily (Fig. 1A). Did these threats, demands, and other efforts at verbal coercion influence ISIS's behavior?

When governments confront rebels, terrorists, or other extremist organizations, verbal conflict is typically dismissed as “cheap talk” (11, 12). Extremists and governments have contradictory goals; one side's gain is the other's loss (13). Both sides have a known incentive to appear strong and resolute in order to extract concessions from the other (14–16). Bluster, bravado, and other verbal actions are essentially costless—and thus indistinguishable from empty threats. According to this conventional wisdom, because verbal attacks are not a credible signal of an actor's strength or willingness to fight, they have no effect on a target's behavior.

Yet, high-resolution event data show that ISIS's attacks on civilians correlated with verbal attacks by foreign governments (Fig. 1B and C). We present results from an extensive analysis of these data using Bayesian structural vector autoregression (B-SVAR) models. The analysis indicates that verbal conflict initiated by governments not only failed to deter ISIS's attacks on civilians but may have increased the likelihood of those attacks. Quantitative and qualitative evidence is consistent with a *credibility deficit* on the part of ISIS. Verbal conflict reinforced ISIS's perception that foreign governments did not take it seriously and did not consider its demands credible. This credibility deficit incentivized ISIS to conduct increasingly violent attacks in an effort to strengthen its reputation. Overall, the results suggest that, by contradicting the organization's perceived reputation, verbal conflict with ISIS perversely encouraged the very actions it was meant to deter. While “backlash” effects from counterterrorism strategies are well known (11, 17), this research is the first, to our knowledge, to quantify the potential negative repercussions of verbal actions, and to show that verbal attacks may provoke terrorists into committing further acts of violence. More generally, our findings point to a need for closer scrutiny of the trade-off between material and verbal conflict.

## Data

We used data from the Integrated Crisis Early Warning System (ICEWS) to model interactions between ISIS and governments (18). Below, we use this same dataset to model additional groups. ICEWS is a machine-coded event dataset, derived from global news sources, that covers daily interactions among national, subnational, and transnational political actors, and is the highest resolution dataset of its kind. Each observation includes (i) a sender, (ii) a target, and (iii) an event type. Senders and targets may be governments, militaries, civilians, rebels, terrorists, or various other political actors. Event types consist of over 270 unique codings, based on the long-established CAMEO coding ontology (10), which cover a range of conflictual and cooperative interactions.

We extracted all observations that identify the Islamic State as either a target or a sender for the time period from 2014 January 1 through 2020 April 30. We then derived five variables. The first two variables measure actions by foreign governments toward ISIS. *Verbal conflict* is a daily count of verbal conflict events directed by any government (sender) against ISIS (target), where “verbal conflict” is defined as any event that involves investigations, demands, condemnations, rejections, overt threats, or reductions in relations. *Material conflict* is a daily count of events involving conventional uses of force, such as airstrikes or ground attacks, directed by any foreign government or military against ISIS.

The last three variables measure ISIS's actions toward various targets. *Attack civilians* is a daily count of events involving coercion, assault, armed physical attacks, and uses of unconventional mass violence directed by ISIS toward any unlawful civilian target, including educational institutions, journalists and media, businesses, and individuals. *Attack militaries* is a daily count of events involving any use of force, conventional or unconventional, by ISIS against formal militaries. *Make threats* is a daily count of overt threats by ISIS toward any target.

These five variables encompass the full range of government actions toward ISIS and potential ISIS responses. They are also generalizable to other extremist groups. SI Appendix A summarizes ICEWS in detail, discusses data validity, and lists the specific CAMEO event and sector codes used for each of the five variables. SI Appendix F considers alternate data sources.

## Main results

**Fig. 2.**
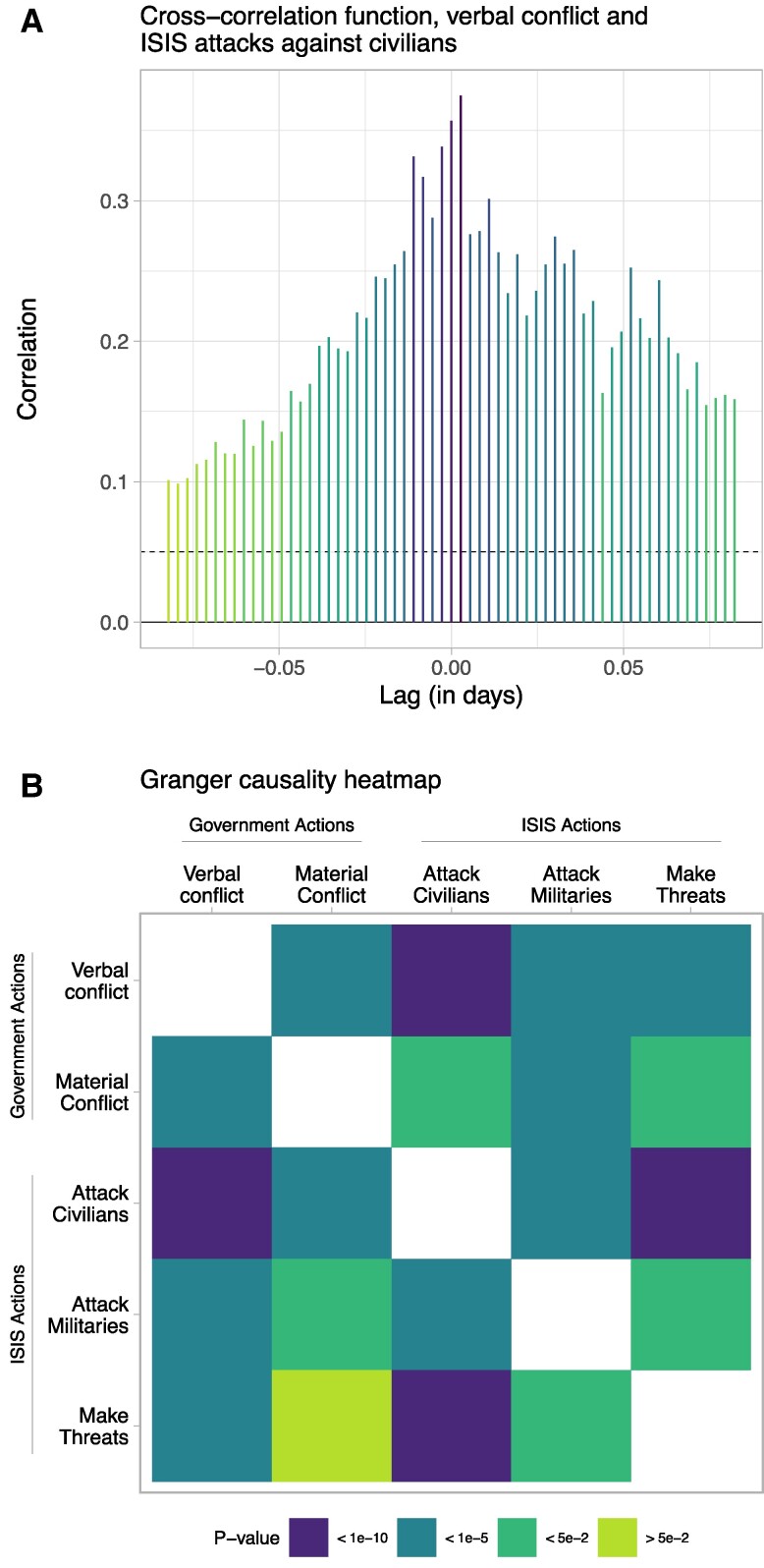
Relationship between *verbal conflict* and *attack civilians*. In (A), the spikes in the CCFs indicate the statistical significance of a given lag or lead. Negative values on the *x*-axis indicate leads, where verbal conflict follows ISIS attacks, and positive values indicate lags. In (B), cells represent pairwise Granger causality test results, with row variables as causes and column variables as responses. Cell color corresponds to statistical significance in *P*-values. Both sets of results show that although, as expected, *attack civilians* frequently precedes or Granger-causes *verbal conflict* initiated by foreign governments, *verbal conflict* also significantly precedes or Granger-causes *attack civilians*. Full results in Table S2.

We are specifically interested in how *verbal conflict* affects *attack civilians*. We first calculated cross-correlation functions (CCFs), which provide preliminary insight into “relations that may occur within and between time series at various lags” (19). The CCFs suggest that a dynamic relationship exists between verbal actions by foreign governments and ISIS attacks on civilians (Fig. 2A). Unsurprisingly, verbal conflict often follows attacks on civilians, consistent with the common practice of governments issuing public statements in response to terror attacks (20, 21, 22). However, the CCF results also show that lags of *verbal conflict* correlate positively with *attack civilians*, which suggests, contrary to existing literature, that verbal attacks by governments may lead extremists to engage in increased violence toward civilians. See SI Appendix B for further discussion and Fig. S1 for the full set of CCFs.

Because governments and ISIS strategically choose among these actions while also responding to one another's behaviors, their interactions are highly interdependent (23). The full set of variables must therefore be modeled as an endogenous system. We used vector autoregressive (VAR), multiequation time-series models to explore the joint dynamics of government actions and ISIS responses (24). These models have the following general reduced form VAR matrix structure:


(1) $$Y_{t}=\sum_{\;j=1}^{J}A_{j}Y_{t-j}+\epsilon_{t}\;\;\;t=1,\;\ldots ,\;T,$$

where *Y_t_* is an *n ×* 1 vector of the variables of interest at time *t* and *A_j_* is an *n × n* matrix of coefficients for the lagged endogenous variables $Y_{t-j}$ at lag *t* − *j*. We tested the data for seasonality and included exogenous seasonal regressors where needed, and conducted tests to ensure stationarity. VAR lag length testing was performed in accordance with the data-driven procedure in Brandt and Williams (24) (Table S1).

Based on the diagnostics, we implemented a series of Granger causality tests (Fig. 2B), which support the findings from the CCFs. Verbal conflict initiated by foreign governments Granger-causes ISIS attacks on civilians at a high level of statistical significance (first row, third column, Fig. 2B). As with the CCFs, the Granger results also show a significant and expected reverse effect of *attack civilians* on *verbal conflict* (third row, first column, Fig. 2B). In SI Appendix G, we consider potential confounding due to this reverse effect. SI Appendix B summarizes the VAR model, lag length testing, CCFs, and Granger causality tests.

### B-SVAR model

While the above tests point toward a causal relationship between verbal conflict and ISIS attacks, inherent features of the data complicate the task of estimating this relationship precisely. All five variables are serially correlated and mutually dependent. Further, the “treatment” of interest—verbal attacks initiated by foreign governments—is nonbinary and repeated, with effects that likely endure over multiple time periods. We must estimate a causal chain or “treatment path” for verbal attacks as their effects play out over time and across interdependent outcomes (25–27). Multivariate models are an effective approach that takes into account reciprocal effects and endogenous outcomes (28, 29) (SI Appendix C.1). Such models have shown particular promise in studying relations between international actors (30, 31).

We implemented a B-SVAR approach (24, 28). This model treats all five variables as endogenous to one another, which allows us to estimate bidirectional, contemporaneous, short-term, and long-term effects. A single equation, containing the lagged and contemporaneous values of all variables in the system, is included for each endogenous variable. The structural component of the SVAR allows us to impose specific expectations on how the variables in the model influence one another, which in turn allows us to assess competing causal arguments, derived from distinct theories of extremist political violence and government actions. See SI Appendix C.3 for further details on B-SVAR models.

By accounting for temporal autocorrelation and structurally identifying contemporaneous dependence, SVAR models are able to “extract sources of exogenous variation” from otherwise endogenous variables (25). This exogenous variation, commonly referred to as a “shock,” triggers a specific treatment path and thus acts as a randomly assigned treatment (32). SVARs use impulse response functions (IRFs) to quantify the effect of shocks in one variable on other variables, estimating “dynamic causal effects” (33). Because IRFs provide insight into both short- and long-term effects, they provide “a richer view of the relationship between the shocks and the variables in the system than a single treatment effect” (25).

Given the complexity of the data, the B-SVAR model is the most appropriate method to assess competing theoretical arguments and isolate potential causal effects. SVARs offer substantial protections against unobserved confounding. Importantly, SVAR analysis does not focus on static correlations among variables as in traditional regression analysis. Rather, the model assesses the *responsiveness* of the system to exogenous shocks in one or more variables. While there are many factors that influence ISIS attacks—territory, funding, recruits, etc.—these are typically large-scale factors that may affect the magnitude or frequency of ISIS attacks but do not obviously affect the responsiveness of ISIS to unexpected government actions. The exclusion from the model of factors that influence ISIS behavior does not necessarily confound the analysis. In order for excluded factors to be confounders, they must be systematically correlated with both daily unexpected changes in government actions and subsequent daily changes in ISIS behavior (34). Although unobserved confounding always remains possible in models using observational data, controlling for numerous potential confounders does not significantly alter the estimates for our main quantities of interest (SI Appendix F). See SI Appendix C.2 for further discussion of causality in B-SVARs.

We tested six competing theoretical arguments, each with unique sets of structural identifications, derived from established literature on conflict, deterrence, and counterterrorism. Of these, we focus on what we term the *credibility model*, which produced the strongest fit. As discussed below, this model represents the theoretical claim that ISIS viewed verbal conflict initiated by governments as reflecting a severe underestimation of the organization's capabilities, and that ISIS subsequently deployed attacks on civilians as a strategy for credibly signaling strength and resolve. SI Appendix C.5 discusses competing causal arguments and the structural analysis. Structural identifications that prioritize other causal pathways, such as those involving government reactions to ISIS atrocities, worsen model fit.

The final B-SVAR model has the following matrix form:


(2) $$A_{0}Y_{t}+\sum_{\;j=1}^{p}A_{j}Y_{t-j}=Z_{t}+\epsilon_{t}\;\;t=1,\ldots ,\;T\;.$$

As in Eq. 1, the endogenous variables at time *t* are in the 5 *×* 1 vector *Y_t_*, while *A_j_* is a 5 *×* 5 matrix of the structural coefficients for the lagged endogenous variables $Y_{t-j}$ at lag *t*  *−*  *j*. The contemporaneous expectations, or structural identifications, are defined in 5 *×* 5 restriction matrix *A*_0_. This approach enables the relationships between the endogenous variables (*Y_t_*) to be included both in contemporaneous (*A*_0_) and in lagged form (*A_j_*). *Z_t_* is a vector containing the intercept, and *ɛ_t_* is a 5 *×* 1 vector of normal independent and identically distributed structural shocks. The model includes a number of Bayesian priors, which were selected following prior evaluation testing (SI Appendix C.4).

### B-SVAR estimates

B-SVAR models enable the tracing of shocks—contemporaneous changes, measured in Sds—as they enter the system of equations. In this case, shocks are actions by or toward ISIS. The model tracks both the direction and speed of the response in some variables to shocks in others, which provides estimates of the immediate and continual effects of ISIS and government actions.

**Fig. 3.**
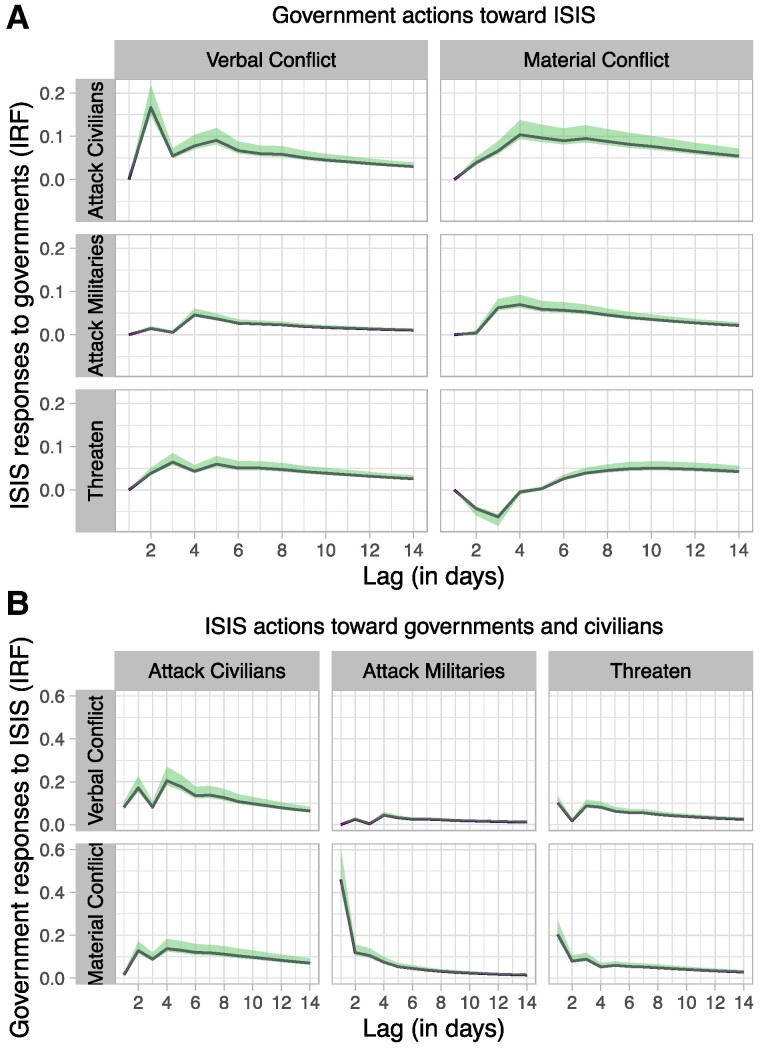
(A) Estimated the effect of government strategies on ISIS behavior. (B) Estimated effect of ISIS behavior on government strategies. *Y*-axis shows IRFs for positive shocks to the system of equations. Error bands are 99% posterior pointwise credible intervals around the median estimates, computed using eigenvector decomposition of the full stacked responses over 2 weeks. Responses are based on one Sd shocks. See SI Appendix D for full results and SI Appendix E for convergence diagnostics.

The full set of estimated IRFs includes 150 responses—25 per model across six structural identifications. We focus here on a subset of those results: ISIS responses to government actions, as well as government responses to ISIS attacks, as estimated in the credibility model (Fig. 3). The results corroborate our initial findings. We again find an expected response by governments to ISIS atrocities (Fig. 3B). At the same time, and contrary to existing literature, we find that ISIS responded strongly to verbal conflict, and this response predominantly took the form of attacks on civilians (Fig. 3A). These attacks peak in severity 2 days after a verbal conflict event and endure for another 2 weeks. While we find evidence that ISIS also attacked civilians in response to material conflict—and, further, that ISIS frequently turned to threats and attacks on militaries—we observe the largest impulse response function (IRF) in the response of the *attack civilians* variable to shocks in the *verbal conflict* variable.

The estimates in Fig. 3 are based on 1-Sd shocks. To understand their substantive significance, we convert the estimates to numeric values and compute the peak effect or largest response. The peak effect, observed at the 2-day mark, translates to one additional attack on civilians for a shock equivalent to 3.14 verbal attacks by foreign governments (Table 1). This observed 3:1 ratio is by far the largest magnitude effect in the main analysis. By contrast, the peak effect of *material conflict* translates to one additional attack on civilians for every 9.98 material attacks—a nearly 10:1 ratio. The substantive effects of verbal and material conflict on the other two outcomes of interest are even weaker. We also calculated the reverse effect of *attack civilians* on *verbal conflict*, which reflects governments’ verbal responses to ISIS attacks. We find that it takes a shock equal to 9.30 attacks against civilians to see one verbal attack in response by governments. That is, while governments do indeed respond to terrorist violence by engaging in verbal attacks, this reverse effect is substantively smaller than the primary effect of *verbal conflict* on *attack civilians* (SI Appendix G).

**Table 1.** Peak observed effect of verbal and material conflict.

Potential ISIS actions	# Verbal conflict events	# Material conflict events
Attack civilians	3.14	9.98
Attack militaries	32.20	41.67
Make threats	12.62	31.80

Cells show the number of *verbal conflict* events and *material conflict* events associated with one additional event in each of the three outcomes of interest. Smaller values indicate effects of greater magnitude.

Verbal conflict appears to be uniquely powerful in triggering violence toward civilians. For perspective, during the height of its activities, between 2013 and 2016, ISIS's terror attacks in Iraq and Syria averaged 6.4 deaths per attack (35). There is a clear opportunity cost associated with verbal conflict, denominated in losses of civilian lives.

## Discussion

Although ISIS controlled territory and operated as a quasi-state, it heavily utilized terrorist strategies, i.e. threats or uses of violence against civilians, governments, infrastructure, and other unlawful targets in order to influence an audience and achieve political goals (36). Scholars have debated whether ISIS should be considered a “terrorist organization” as such, but this debate is immaterial to our theory and analysis. In its use of terror attacks as a strategy for achieving political goals, ISIS is similar to Fuerzas Armadas Revolucionarias de Colombia (FARC), Partiya Karkeran Kurdistan (PKK), and numerous other highly organized extremist groups that have espoused ambitious objectives, controlled large swaths of territory, provided public services, and utilized conventional military tactics alongside terror attacks. Our analysis does not assume that ISIS is a terrorist organization; it assumes only that ISIS is an extremist political organization whose interests conflict with the interests of governments. The arguments developed here are generalizable to a variety of actors, from terrorists and rebels to insurgencies and nonstate armed groups.

As in other forms of political conflict, interactions between governments and extremists resemble a two-player bargaining game. Each side strategically responds to the other's actions and statements, such that their decisions are interdependent (23). Importantly, one or both sides lack crucial information about the other's capabilities (12, 37). Governments are particularly uncertain of whether extremists have the means and the will to inflict pain and suffering on civilian targets (14, 15, 23, 38).

Extremists use terror attacks to signal strength and resolve to an uninformed target audience (11, 14, 39, 40). These attacks expend blood and treasure, invite indiscriminate retaliation, risk loss of legitimacy, and deliberately increase the stakes of political confrontations (39). By showing a willingness to incur costs, terror attacks function as costly signals that separate weak, irresolute organizations from organizations that are determined to achieve their goals and materially capable of doing so (12, 41).

In practice, however, costly signals often fail (42). The target may doubt the sender's sincerity (43). Misperception and cognitive biases may distort the target's interpretation of a signal (44, 45, 46, 47). The target's assessment of the sender's credibility may be influenced by personal impressions (48), emotions (49), or political dispositions (50).

Evaluating the efficacy of one's signals necessarily involves “second-order beliefs,” or beliefs about the beliefs of others (51). When audiences treat an organization as if it is strong and determined, that organization's beliefs regarding its reputation respond accordingly (52). As illustrated by research on how terrorist organizations manage their reputations (53), negotiate with governments (54, 55), and influence public opinion (56, 57), extremist groups routinely monitor the impact of their actions on target audiences. By observing the government's choice of strategies, extremists draw inferences about their reputation and determine whether terror attacks yield the desired effect.

Because governments and extremists both have strong incentives to misrepresent their capabilities (15, 58), verbal conflict is merely “cheap talk” and should have no effect on either actor's behavior (11). Yet, experimental and observational studies find that cheap talk affects strategic behavior in many contexts, including entry-deterrence experiments (59), prisoner's dilemma experiments (60), civil wars (61), and diplomatic communications (62, 63).

Further, verbal attacks often backfire, inflicting unintended consequences (64, 65). In negotiation and mediation, threats encourage targets to harden their positions or allocate more resources to the dispute (66, 67). In bargaining experiments, targets of threats often respond with belligerence, hostility, and increased resistance (68, 69). Reputation costs—or “loss of face”—are a key mechanism behind this response (70, 71). Threats, demands, denunciations, and similar actions reveal that the sender believes the target is sufficiently weak as to be deterred by bluffs, bravado, and mere cheap talk.

Verbal conflict thus undermines an extremist group's efforts at establishing a credible reputation for strength and resolve. The result is a *credibility deficit.* The government's use of costless signals reveals to extremists that their own signals lack credibility, thus compelling a revision of the organization's second-order beliefs regarding its reputation. This credibility deficit incentivizes increasingly extreme and costly attacks, where extremists attempt to unequivocally signal strength and resolve in the face of government strategies that question their capabilities. By contradicting the organization's assessment of its own reputation, verbal conflict encourages precisely the actions it is meant to deter.

Qualitative evidence shows that these mechanisms were widely apparent in ISIS's activities. As with other contemporary extremist groups, ISIS used digital media and other communication platforms to carefully tailor its reputation to targeted audiences—for example, by spreading information about the organization's history, goals, and perceived political challenges (72, 73, 74, 75). ISIS specifically cultivated a reputation as an ideologically committed organization, willing to engage in extreme brutality in order to intimidate local populations and extract concessions from foreign governments (36). A key element of this strategy was ISIS's online magazine, *Dabiq*, published from mid-2014 to mid-2016. A recurring section of *Dabiq* titled “In the Words of the Enemy” reproduced statements from foreign leaders, security analysts, and journalists that, in the eyes of ISIS, affirmed the organization's fearsome reputation. Examples include a statement from Israeli Prime Minister Benjamin Netanyahu that “ISIS has got to be defeated. […] It is dangerous, no question,” and a statement from US Secretary of Defense Chuck Hagel that the United States has “never seen an organization like [ISIS] that is so well-organized, so well-trained, so well-funded, so strategic, so brutal, so completely ruthless. […] You blend all of that together, that is an incredibly powerful new threat” (76).

These examples further illustrate ISIS's practice of assessing the efficacy of its reputation-building efforts by closely monitoring the responses of global audiences, especially governments and political leaders. ISIS monitored its reputation so fervently that it often highlighted or responded to seemingly minor statements. For example, in the third issue of *Dabiq*, ISIS detailed an attack on a Syrian Army officer who was “killed after he boasted on television of false victories” against the organization (77).

Analysis of historical data further shows that ISIS leadership interpreted threats, demands, denunciations, and other acts of verbal conflict as underestimations of the organization's capabilities, and frequently justified civilian attacks on that basis. Following the beheading of US journalist James Foley in 2014 August, President Obama denounced ISIS as “cowardly,” described it as having “no ideology of any value to human beings” and “no place in the 21st century,” and threatened to “do what's necessary to see that justice is done” (78). In a subsequent issue of *Dabiq*, ISIS declared that its demands for a prisoner exchange had been “arrogantly ignored” by the Obama Administration and that “Foley's Blood is on Obama's hands” (77). Shortly after the United States again rejected its demands, ISIS beheaded US–Israeli citizen Steven Sotloff. Similarly, when Japanese Prime Minister Shinzo Abe promised $2 million in aid to countries fighting the Islamic State, ISIS leadership demanded a contribution in kind. After Japan refused, ISIS decapitated two Japanese prisoners, declaring that Abe had failed to “[heed] the warnings of the Islamic State” (79).

In 2016 June, Iraq's security forces and government-allied militias criticized ISIS's defensive capabilities in the fight for Fallujah, asserting that the Islamic State's “defensive lines collapsed” and that it “did not fight seriously” (80). A week later, ISIS orchestrated a bombing attack that killed over 300 civilians in the Karada neighborhood of Baghdad, which ISIS leadership described in *Dabiq* as “terrorizing, massacring, and humiliating” its enemies (81). When the international community publicly committed to the organization's “defeat and destruction,” ISIS responded by targeting civilians in France, the United Kingdom, Belgium, Australia, Germany, and other countries that had pledged to join the fight. After ISIS lost control of the Iraqi city of Ramadi, the Prime Minister of Iraq claimed that ISIS was “collapsing” and promised to liberate the rest of Iraq from “the terrorist Daesh gang” (82). ISIS responded by launching coordinated retaliatory attacks against civilians in Iraq, Syria, and Libya.

Together, these examples reflect the mechanisms behind credibility deficits. ISIS combined brutal treatment of civilian populations with digital media to convince global audiences of its strength and resolve. ISIS assessed its reputation by monitoring the responses of those audiences, especially government actors. Finally, ISIS perceived demands, denunciations, threats and other forms of verbal conflict as underestimating the organization's strength and resolve, and it engaged in further acts of extreme violence in an effort to solidify its reputation, enhance the credibility of its threats, and increase the odds that its demands would be met (83).

Importantly, our argument does not imply that ISIS attacks were driven solely, or even primarily, by credibility. Like other extremist groups, ISIS used attacks on civilians in part to exert local control and outbid competing organizations (11, 84, 85). Such strategies affect the aggregate frequency or magnitude of ISIS attacks, and they depend on influences, such as organizational capacity, that fall outside the scope of our analysis. By contrast, we ask whether ISIS attacks were *responsive to* verbal conflict initiated by foreign governments—a relationship distinct from ISIS's overall willingness to attack civilians. Our argument also does not imply that ISIS derived no strategic benefits from verbal conflict. Indeed, verbal attacks by powerful governments may have increased the organization's public exposure (86). Yet, if ISIS viewed verbal conflict as strictly beneficial, then it would have no incentive to respond to such conflict by harming civilians, and we should not observe an empirical relationship between the two phenomena. SI Appendix F–H consider alternative explanations, such as “clash of civilizations” arguments, efforts at territorial control, the influence of exogenous factors like ISIS's financial resources and number of active fighters, and the possibility that the estimated effect of *verbal conflict* on *attack civilians* is an artifact of multiday military campaigns, follow-up attacks, or routine government responses to terrorist violence.

## Additional results

Credibility is not directly observable but must be assessed indirectly—as in the above examples—by observing the behaviors of actors (12, 87, 88). The logic of a credibility deficit offers multiple testable implications. First, we disaggregated the data by time period. Reputations take time to solidify and are most uncertain when an actor first emerges (89, 90). If ISIS indeed used attacks on civilians to signal strength and resolve to audiences, then we should observe that those attacks were most responsive to verbal conflict in the initial months of ISIS's emergence, when its reputation was not yet established and audiences were unsure of its capabilities and intentions. By contrast, if ISIS used civilian attacks solely to suppress resistance or exert territorial control, and/or if the attacks were simply a reflection of ISIS's fanatical ideology, then we should observe that attacks are generally unresponsive to verbal conflict irrespective of time period (see SI Appendix H for further discussion).

**Fig. 4.**
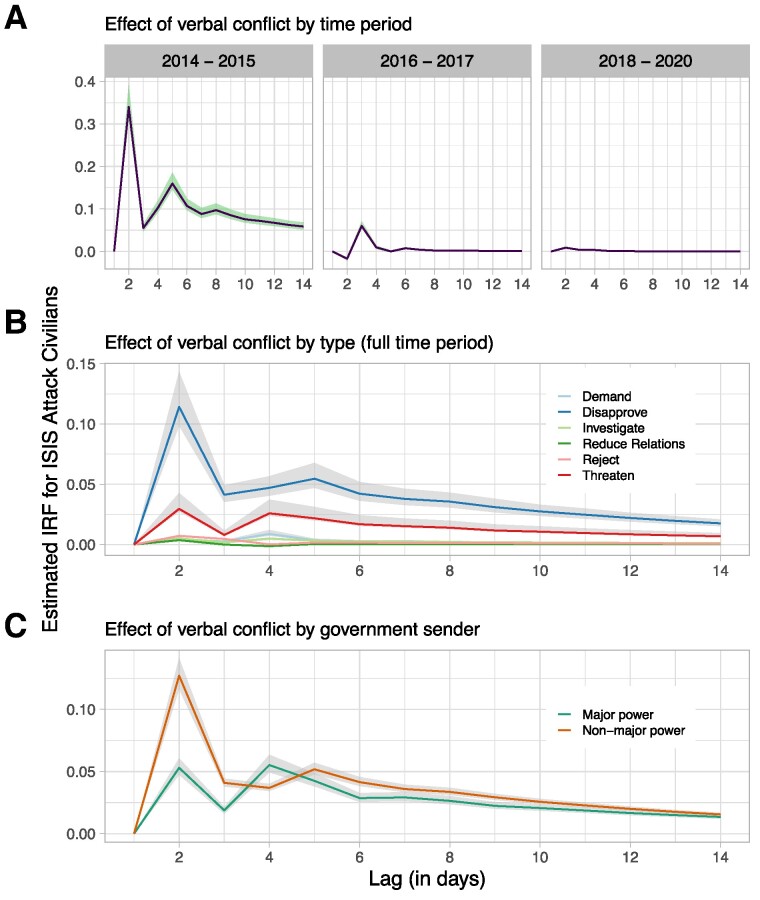
Estimates from B-SVAR models on disaggregated data. Error bands are 99% credible intervals. A) Effect of *verbal conflict* on *attack civilians* by time period. 2014–2015 covers the rise of ISIS and the establishment of the Islamic State. 2016–2017 covers ISIS's initial decline in Syria and especially in Iraq; losses of Fallujah, Raqqa, and Mosul; and the loss of up to 90% of foreign recruits and 75% of online media presence. 2018–2020 covers the containment of ISIS, the loss of its remaining territory, and the removal and replacement of its leader. B) Effect of verbal conflict in six separately estimated models, using constituent metrics of *verbal conflict*, for the full 2014–2020 time period. C) Effect of verbal conflict initiated by major powers versus nonmajor powers, where “major power” refers to permanent members of the UN Security Council, plus Germany and Japan.

Re-estimating the B-SVAR model on separate time periods reveals that the effect of *verbal conflict* on *attack civilians* was strongest during the 2014–2015 period, which includes the rise of ISIS and the metastasis of the organization across Iraq and Syria (Fig. 4A). The peak observed effect here approaches a 1:1 ratio, with ISIS launching one additional attack on civilians for every 1.74 verbal attacks by foreign governments. During the following period, 2016–2017, the effect weakened substantially; each attack on civilians was associated with 11.62 verbal attacks. As the organization's reputation solidified, and as a diminution in capabilities lessened the gap between ISIS's reputation and the actions of foreign governments, the need for costly signals declined. Finally, in the 2018–2020 period, when ISIS switched to a low-cost campaign and focused on stanching territorial and leadership losses, the organization's treatment of civilians was largely unresponsive to verbal conflict.

Next, we disaggregated *verbal conflict* into its six constituent metrics, each of which reflects a specific type of verbal conflict (SI Appendix A.3). We expect ISIS to respond weakly, if at all, to reductions in formal relations and launching of investigations, as both actions are routine policies during conflict and do not affect perceptions of credibility. We anticipate a stronger response to demands and rejections. While both actions are standard elements of bargaining processes, if governments levy demands that are too strong, and/or if they reject counter-demands that ISIS views as reasonable, then ISIS may infer that it suffers from a credibility deficit. We expect ISIS to react most strongly to explicit threats and to acts of disapproval or denunciation. As the above examples illustrate, ISIS viewed such actions as direct affronts to its credibility.

The estimates generally reflect these expectations (Fig. 4B). We observe the strongest effect for disapprovals, which lead to one additional attack on civilians for every 3.41 acts of disapproval (Table 2), consistent with ISIS's perception that these actions particularly diminished its reputation. We also find a strong effect for demands and threats, and a moderate effect for rejections. As expected, ISIS responded most weakly to reductions in relations and investigations.

**Table 2.** Observed peak effect of six constituent metrics of *verbal conflict* on *attack civilians*.

Reduce relations	15.44
Investigate	15.02
Reject	11.24
Threaten	5.88
Demand	5.05
Disapprove	3.41

Cells show the number of events associated with one additional attack on civilians. Smaller values indicate effects of greater magnitude.

Next, we differentiated between verbal attacks initiated by major powers—conventionally defined as the permanent five members of the UN Security Council, plus Japan and Germany (91)—and those initiated by nonmajor powers. Extremists are more likely to suffer a credibility deficit if they are verbally attacked by powerful, highly visible governments, and they are more likely to monitor such governments for statements or actions that undermine the group's reputation. As expected, ISIS was more immediately responsive to verbal attacks from powerful governments than from weaker governments (Fig. 4C), consistent with the credibility argument.

Finally, we considered the generalizability of this finding to other high-profile extremist groups. We applied the B-SVAR model to AQI, the central participant in the sectarian violence that dominated Iraq in the mid-2000s. We focus on the 2004–2006 period, which covers the organization's emergence and eventual termination. We also applied the model to Boko Haram, a jihadist organization headquartered in Northeastern Nigeria whose insurgency resulted in over 20,000 civilian deaths. We focus on the period 2009–2014, which extends from the Boko Haram uprising in 2009 July until just prior to the organization's transformation in early 2015.

**Fig. 5.**
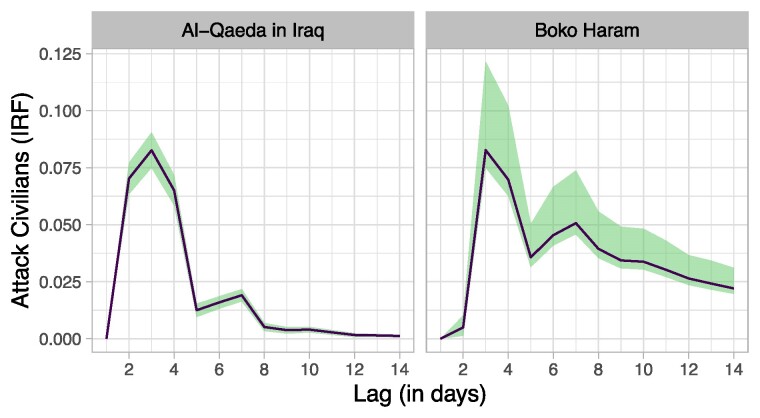
Effect of verbal conflict with AQI and Boko Haram on attacks against civilians. Results are from two separately estimated B-SVAR models. Panels plot the impulse response function between *verbal conflict* and *attack civilians*. Error bands are 99% credible intervals.

The results show the same fundamental relationship between *verbal conflict* and *attack civilians* as we detected with ISIS (Fig. 5). Within 2 to 5 days of a verbal attack by a foreign government, the organization responds by attacking civilians. As in our other models, this estimated relationship persists even after we account for the known reverse tendency of governments to verbally respond to extremist violence. Substantively, the estimates in Fig. 5 translate, in the case of AQI, to one additional attack on civilians for every 7.80 verbal attacks, and in the case of Boko Haram, to one additional attack on civilians for every 2.76 verbal attacks. For AQI this effect dissipates within a week, while for Boko Haram it endures well beyond 2 weeks.

Like ISIS, both organizations were initially not well known. A series of high-profile attacks—many of them suicide bombings, often characterized by extreme losses of civilian life—quickly established their respective reputations. Attempts at verbally coercing these organizations had the opposite effect. Verbal conflict antagonized the terrorists, undermined their efforts at reputation building, and perversely created incentives to launch further attacks. Boko Haram, which lacked the same terror network connections as AQI and thus faced a particular risk of credibility deficits, was especially responsive to these incentives. Further, Boko Haram was not as eager to claim credit for its attack as AQI and ISIS were (92), which suggests that our model's expectations hold even when attribution of attacks is a concern. Nonetheless, as with other bargaining approaches to terrorism, our model's expectations may not be realized in situations where the affiliation of attackers is opaque (93). Overall, the generic nature of the theory and derived variables, combined with the results for additional groups, suggest that the findings here are generalizable well beyond ISIS.

Research on counterterrorism has found that although proactive measures—such as destroying training camps or killing/capturing members—generate greater public benefits than passive or defensive measures, governments are reluctant to incur the costs and risks of engaging extremists directly and thus are far more likely to implement defensive measures than proactive ones (94). However, the results presented here suggest that the conventional view of verbal conflict as costless cheap talk may be incorrect. At the same time, our results do not imply that governments should prioritize material conflict over verbal conflict. Militarized actions against extremists involve a separate set of humanitarian considerations, such as unintended civilian casualties (“collateral damage”) and troop fatalities, which fall outside the scope of the present analysis but require attention from leaders and policymakers. Rather, the main insight of the present analysis is that while verbal conflict against terrorists does not carry the same financial, military, and political costs as material conflict, it may inflict a significant, measurable cost in terms of civilian lives.

## Supplementary Material

pgae437_Supplementary_Data

## Data Availability

The data underlying this article are available at https://dataverse.harvard.edu/dataverse/icews and are publicly accessible.
