# Supplementary material for: Verbal attacks on terrorist groups increase violence against civilians
Source: PNAS Nexus. 2024 Oct 2;3(10):pgae437. doi: 10.1093/pnasnexus/pgae437 (PMC11477982; doi:10.1093/pnasnexus/pgae437)
Supplement: pgae437_Supplementary_Data [file pgae437_supplementary_data.pdf]

Supporting Information

"Verbal attacks on terrorist groups increase violence against civilians"

Contents

A Data Sources and Coding Guidelines **2**

A.1 ICEWS Validity . . . 2

A.2 Coding Rules . . . 3

A.3 ICEWS Event Codes . . . 4

B Initial Models **7**

C Bayesian Structural VAR Model **10**

C.1 Univariate vs Multivariate Models . . . 10

C.2 Causality Claims and Intervening Variables . . . 11

C.3 Why use a Bayesian model . . . 11

C.4 Choice of Priors . . . 12

C.5 Structural Identification . . . 14

D Discussion of Final Results **19**

E Convergence Diagnostics **22**

F Additional Models and Robustness Checks **25**

F.1 Analysis of data subsets . . . 25

F.2 Analysis with exogenous controls . . . 27

F.3 Analysis of ISIS-inflicted fatalities . . . 27

G Twoway Influence between Verbal Conflict and ISIS Attacks **30**

G.1 Additional empirical analysis . . . 31

H Further Discussion **35**

A Data Sources and Coding Guidelines

Our main data source, the Integrated Crisis Early Warning System (ICEWS), is "a comprehensive, integrated, automated, generalizable, and validated system to monitor, assess, and forecast national, sub-national, and international crises" [1]. In short, ICEWS exhaustively catalogs "who, did what, to whom, when, and where" on a global scale. While empirical research in international relations often utilizes data aggregated to the annual (yearly) level, event data are more appropriate for research questions that involve specific types of political behavior over short time spans [2, 3]; ICEWS is, to our knowledge, the most fine-grained time-varying event dataset available. ICEWS includes nearly 20 million unique events covering hundreds of political, social, and economic actors since 1995. It especially emphasizes the "complexity of interactions" among governments, people, and nonstate actors, with the goal of identifying "generalizable patterns" in those interactions [1]. These features make ICEWS ideally suited for the present analysis. Research using ICEWS has appeared in leading political science and international relations journals [4, 5, 6, 7, 8], as well as general science journals [9, 10, 11]. A large majority of this work focuses on political violence at the subnational and transnational levels.

Although there exist other datasets that contain ISIS-related events, these other sources have important limitations. For example, the Armed Conflict Location & Event Data (ACLED) project's coverage of Iraq and Syria begins in 2016 and 2017, respectively—long after ISIS emerged as a regional threat and accumulated territory [12]. The disaggregated data provided by the Uppsala Conflict Data Program (UCDP) are restricted to "lethal violence" and thus only include ISIS attacks that resulted in fatalities [13]. (Note, however, that we use the UCDP data below to assess the robustness of our findings to counts of fatalities instead of counts of attacks.) The Global Terrorism Database (GTD) includes only actions by ISIS that might qualify as terrorism and does not provide information on broader interactions between ISIS and governments [14]. ICEWS is thus the most appropriate dataset for our analysis.

A.1 ICEWS Validity

The limitations of automated event data are well known, such as difficulty in capturing the context of news stories, changes in the resource corpus over time, and reliance on antiquated dictionary-based pattern matching. ICEWS addresses these limitations more effectively than competing resources—for example, by relying on a stable resource corpus and incorporating machine-learning methods [10].

Validation of text-based data is an area of ongoing research [15]. While attempting to validate each of the millions of data points in ICEWS would of course defeat the purpose of automated event coding, existing analyses suggest high internal validity. For example, around 80% of likely protest events are coded as such (compared to only 21% of events in a competing dataset, GDELT) [10]. Comparison of hand-coded to machine-coded data shows coding accuracy of 74–85% in randomly selected ICEWS events [16].

Numerous studies also indicate high external validity. One study finds strong correspondence between ICEWS-based forecasts and real-world events in Colombia and Venezuela [17]. Another finds a strong correspondence between ICEWS events and "ground truth" data [18]. Another finds that ICEWS data mirror domestic volatility in Egypt, Turkey, and Syria [16]. Another finds that while correlations between ICEWS and curated ground-truth data sets, such GSR and SPEED,

tend to be low, these correlations strengthen substantially for large upticks in event counts [10]. A particularly important concern with external validity is reporting bias [19]. A thorough comparison of ICEWS data and human-coded textual data finds that "machine-coded data are as valid as the human-coded data" [20].

The practical utility of ICEWS data is substantial. Models using ICEWS data are able to obtain high levels of precision and recall in out-of-sample forecasts of insurgency, rebellion, domestic political crises, ethnic violence, civil war, and dyadic crises—in some cases as high as 80% [10, 21, 22]. Importantly, the event types that have been subjected to the greatest level of scrutiny are precisely the sorts of events in which we are most interested.

A.2 Coding Rules

A typical ICEWS entry includes three crucial pieces of information: (1) the respective "sender" and "target" of an event, which can include any number of domestic, transnational, or international actors or agents; (2) the date on which the event occurred; and (3) the "tone" of the event itself, which ranges from cooperative to neutral to conflictual, and may be either "verbal" or "material."

We implement a set of transparent, rigorous guidelines in extracting usable data from ICEWS. First, we convert ICEWS entries to the standardized event and agent codes specified by the Conflict and Mediation Event Observations (CAMEO) framework—by far the most widely used political event data ontology [23, 24]. We use a modified version of the `text2cameo` algorithm to assign all senders and targets to one of thirteen unique agent codes: government (GOV), military (MIL), rebel (REB), opposition (OPP), political party (PTY), police and internal security (COP), judicial actor (JUD), intelligence service (SPY), media (MED), educational actor (EDU), business (BUS), criminal (CRM), or civilian (CVL) [25].

The `text2cameo` algorithm further assigns a unique CAMEO *event code* to each ICEWS observation. The CAMEO ontology includes over 270 such event codes, which indicate the tone of an interaction with a high degree of specificity. For example, event code 1952 corresponds to "Employ remotely piloted aerial munitions," and event code 191 corresponds to "Impose blockade, restrict movement." Each of these 270+ event codes falls into one of twenty larger *root codes.* For example, both event code 1952 and 191 fall under root code 19, "Fight."

At the highest level of aggregation, each of these twenty root codes, as well as the 270+ event codes that comprise them, fall into one of four *quad codes:* verbal cooperation, verbal conflict, material cooperation, material conflict. We use this hierarchy of codes to generate the main variables, as detailed below.

Second, we extract from the full ICEWS dataset all events that involve ISIS as either a target or sender, using the *Source Name* and *Target Name* fields in ICEWS. Applying this filter yields approximately 27,000 unique events. (For the models of Boko Haram and al-Qaeda in Iraq, we instead extract data for those specific actors.)

Third, from the subset of ISIS data we generate five variables that capture governments' actions toward ISIS, and ISIS's actions toward governments, civilians, and others. We define governmental actors broadly to include the incumbent government itself (GOV), formal militaries (MIL), judiciaries (JUD), political parties (PTY), and security forces (COP). We first derive two variables with governments as "sender" and ISIS as "receiver":

- *Verbal Conflict:* A daily count of all government → ISIS events that are considered "verbal conflict" (quad code 3) by the CAMEO ontology. These include all events in root codes 09 ("Investigate"), 10 ("Demand"), 11 ("Disapprove"), 12 ("Reject"), 13 ("Threaten"), and 16 ("Reduce Relations"). See Appendix A.3 for the full list of event codes within each of these root codes.
- *Material Conflict:* A daily count of all government → ISIS events that fall under root code 19, "Fight," in the CAMEO ontology. See Appendix A.3 for the full list of event codes within this root code.

We then generate three variables with ISIS as "sender" and various actors as "target," as follows:

- *Attack Civilians:* A daily count of events within CAMEO root codes 17 ("Coerce"), 18 ("Assault"), and 20 ("Use Unconventional Mass Violence"), directed by ISIS toward either civilians (CVL), educational actors (EDU), media actors (MED), or businesses (BUS). See Appendix A.3 for the full list of event codes within these root codes.
- *Attack Militaries:* A daily count of events within CAMEO root codes 17 ("Coerce"), 18 ("Assault"), 19 ("Fight"), and 20 ("Use Unconventional Mass Violence"), directed by ISIS toward military targets (MIL). See Appendix A.3 for the full list of event codes within these root codes.
- *Make Threats:* A daily count of events within root code 13 ("Threaten"), directed by ISIS toward any actor—civilian, administrative, military, or otherwise. See Appendix A.3 for the full list of event codes within this root code.

Because event data sets may include duplicate entries (for example, from multiple news sources reporting on the same event), we also generate versions of these variables using a deduplication filter. We impose a strict one-a-day filter, which limits each unique sender-target pair to one event per CAMEO event code per day. This filter reduces the number of usable events by about 20%. Importantly, because actor pairs may have multiple interactions within a single day, this filter almost certainly removes some true positives. The unfiltered and filtered variables thus represent opposite extremes. The unfiltered data errs on the side of including all events, even if some of those events are duplicates. The filtered data errs on the side of excluding duplicates, even if some true events are also excluded. We show below that the results from the main paper hold regardless of which version we use. See Figures S6 and S7 in Appendix D, and the corresponding discussion.

A.3 ICEWS Event Codes

The following list summarizes the unique event codes within each of the root codes used to derive the variables for our analysis:

- **09 Investigate:** 090 ("Investigate, not specified below"), 091 ("Investigate crime, corrouption"), 092 ("Investigate human rights abuses"), 093 ("Investigate military action"), 094 ("Investigate war crimes")

- **10 Demand:** 100 (“Demand, not specified below”), 101 (“Demand material cooperation”), 1011 (“Demand economic cooperation”), 1012 (“Demand military cooperation”), 1013 (“Demand judicial cooperation”), 1014 (“Demand intelligence cooperation”), 102 (“Demand for diplomatic cooperation”), 103 (“Demand material aid”), 1031 (“Demand economic aid”), 1032 (“Demand military aid”), 1033 (“Demand humanitarian aid”), 1034 (“Demand military protection or peacekeeping”), 104 (“Demand political reform”), 1041 (“Demand leadership change”), 1042 (“Demand policy change”), 1043 (“Demand rights”), 1044 (“Demand change in institutions, regime”), 105 (“Demand that target yield”), 1051 (“Demand easing of administrative sanctions”), 1052 (“Demanding easing of political dissent”), 1053 (“Demand release of persons or property”), 1054 (“Demand easing of economic sanctions, boycott, or embargo”), 1055 (“Demand to allow international involvement”), 1056 (“Demand de-escalation of military engagement”), 106 (“Demand meeting, negotiation”), 107 (“Demand settling of dispute”), 108 (“Demand mediation”)

- **11 Disapprove:** 110 (“Disapprove, not specified below”), 111 (“Criticize or denounce”), 112 (“Accuse”), 1121 (“Accuse of crime, corruption”), 1122 (“Accuse of human rights abuses”), 1123 (“Accuse of aggression”), 1124 (“Accuse of war crimes”), 1125 (“Accuse of espionage, treason”), 113 (“Rally opposition against”), 114 (“Complain officially”), 115 (“Bring lawsuit against”), 116 (“Find guilty or liable (legally)”)

- **12 Reject:** 120 (“Reject, not specified below”), 121 (“Reject material cooperation”), 1211 (“Reject economic cooperation”), 1212 (“Reject military cooperation”), 1213 (“Reject judicial cooperation”), 1214 (“Reject intelligence cooperation”), 122 (“Reject request or demand for material aid”), 1221 (“Reject request for economic aid”), 1222 (“Reject request for military aid”), 1223 (“Reject request for humanitarian aid”), 1224 (“Reject request for military protection or peacekeeping”), 123 (“Reject request or demand for political reform”), 1231 (“Reject request to change leadership”), 1232 (“Reject request to change policy”), 1233 (“Reject request for rights”), 1234 (“Reject request for change in institutions, regime”), 124 (“Refuse to yield”), 1241 (“Refuse to ease administrative sanctions”), 1242 (“Refuse to ease popular dissent”), 1243 (“Refuse to release persons or property”), 1244 (“Refuse to ease economic sanctions, boycott, or embargo”), 1245 (“Refuse to allow international involvement”), 1246 (“Refuse to de-escalate military engagement”), 125 (“Reject proposal to meet, discuss, negotiate”), 126 (“Reject mediation”), 127 (“Reject plan, agreement to settle dispute”), 128 (“Defy norms, law”), 129 (“Veto”)

- **13 Threaten:** 130 (“Threaten, not specified below”), 131 (“Threaten non-force”), 1311 (“Threaten to reduce or stop aid”), 1312 (“Threaten to boycott, embargo, or sanction”), 1313 (“Threaten to reduce or break relations”), 132 (“Threaten with administrative sanctions”), 1321 (“Threaten with restrictions on political freedoms”), 1322 (“Threaten to ban political parties or politicians”), 1323 (“Threaten to impose curfew”), 1324 (“Threaten to impose state of emergency or martial law”), 133 (“Threaten political dissent”), 134 (“Threaten to halt negotiations”), 135 (“Threaten to halt mediation”), 136 (“Threaten to halt international involvement”), 137 (“Threaten with repression”), 138 (“Threaten with military force”), 1381 (“Threaten blockade”), 1382 (“Threaten occupation”), 1383 (“Threaten unconventional attack”), 1384 (“Threaten conventional attack”), 1385 (“Threaten unconventional mass violence”), 139 (“Give ultimatum”)

- **16 Reduce Relations:** 160 (“Reduce relations, not specified below”), 161 (“Reduce or break diplomatic relations”), 162 (“Reduce or stop military aid”), 1621 (“Reduce or stop economic

assistance"), 1622 ("Reduce or stop military assistance"), 1623 ("Reduce or stop humanitarian assistance"), 163 ("Impose embargo, boycott, or sanctions"), 164 ("Halt negotiations"), 165 ("Halt mediation"), 166 ("Expel or withdraw"), 1661 ("Expel or withdraw peacekeepers"), 1662 ("Expel or withdraw inspectors, observers"), 1663 ("Expel or withdraw aid agencies").

- **17 Coerce:** 170 ("Coerce, not specified below"), 171 ("Seize or damage property"), 1711 ("Confiscate property"), 1712 ("Destroy property"), 172 ("Impose administrative sanctions"), 1721 ("Impose restrictions on political freedoms"), 1722 ("Ban political parties or politicans"), 1723 ("Impose curfew"), 1724 ("Impose state of emergency or martial law"), 173 ("Arrest, detain"), 174 ("Expel or deport individuals"), 175 ("Use repression")

- **18 Assault:** 180 ("Use unconventional violence, not specified below"), 181 ("Abduct, hijack, take hostage"), 182 ("Physically assault"), 1821 ("Sexually assault"), 1822 ("Torture"), 1823 ("Kill by physical assault"), 183 ("Conduct suicide, car, or other nonmilitary bombing"), 1831 ("Carry out suicide bombing"), 1832 ("Carry out vehicular bombing"), 1833 ("Carry out roadside bombing"), 1834 ("Carry out location bombing"), 184 ("Use as human shield"), 185 ("Attempt to assassinate"), 186 ("Assassinate")

- **19 Fight:** 190 ("Use conventional military force, not specified below"), 191 ("Impose blockade, restrict movement"), 192 ("Occupy territory"), 193 ("Fight with small arms and light weapons"), 194 ("Fight with artillery and tanks"), 195 ("Employ aerial weapons"), 1951 ("Employ precision-guided aerial munitions"), 1952 ("Employ remotely piloted aerial munitions")

- **20 Engage in Unconventional Mass Violence:** 200 ("Use massive unconventional force, not specified below"), 201 ("Engage in mass expulsion"), 202 ("Engage in mass killings"), 203 ("Engage in ethnic cleansing"), 204 ("Use weapons of mass destruction"), 2041 ("Use chemical, biological, or radiological weapons")

B Initial Models

As an initial diagnostic, we performed analysis using cross-correlation functions or CCFs. Cross-correlation functions allow us to diagnose temporal relations that might exist in the data. They are "tools for clarifying relations that may occur within and between time series at various lags" [26]. The method is widely used to study various political phenomena [27, 28, 29] and is useful as an initial step to determine temporal relations among variables as either lagging or leading indicators. CCFs analyze variables as a dynamic system, depicting changes as a reaction to or expectation for changes in another variable.

Figure S1 includes a graphical representation of the CCFs for all variables. While the CCFs suggest a number of influences among the variables in the dataset, we find a consistently strong relationship between the two key variables of interest, *Verbal Conflict* and *Attack Civilians* (top row, second column). This result shows not only that, as expected, lags of *Attack Civilians* significantly correlate with *Verbal Conflict*, but that lags of *Verbal Conflict* also strongly correlate with *Attack Civilians*. Note that CCFs only reflect dot-product correlations among arbitrarily specified lags; they cannot determine whether, for example, one direction might be spurious to the other.

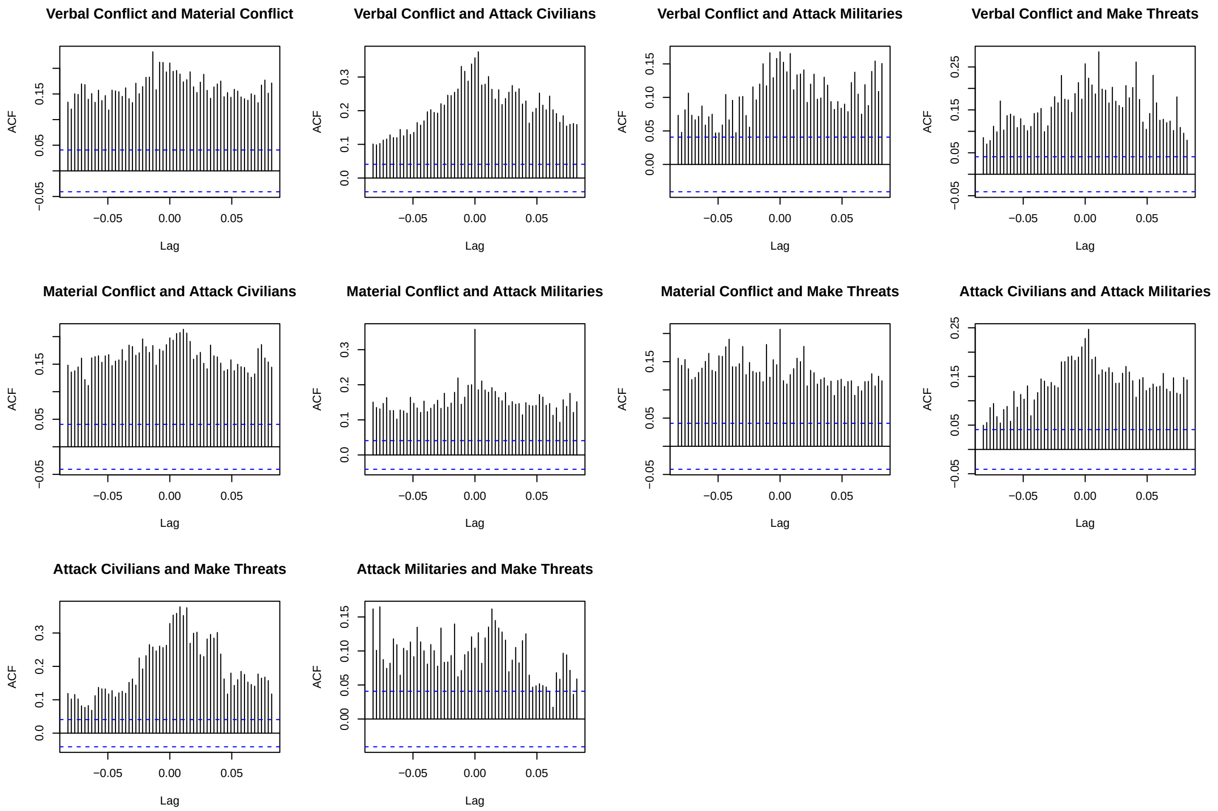

Figure S1: Cross-correlation functions. These results are a function of lag *l*, where negative values on the x-axis represent leads and positive values depict lags. The spikes show the statistical significance of a specific lag/lead relationship. The dashed line is the 95% confidence bound. A lag of zero is the contemporaneous responses (same day).

To further study the joint dynamics of government actions and ISIS responses we fitted a series of vector autoregressive (VAR) models [30]. These are multi-equation time series models that include multiple dependent variables for effects of government actions and ISIS actions. The general reduced

form VAR matrix structure of the model, including the lagged and contemporaneous values of all variables in the system, is:

$$Y_t = \sum_{j=1}^{J} A_j Y_{t-j} + \epsilon_t \qquad t = 1, ..., T, \tag{1}$$

where Y_t is an $n \times 1$ vector of the variables of interest at time t, and A_j is a $n \times n$ matrix of coefficients for the lagged endogenous variables Y_{t-j} at lag $t - j$.

VAR models require lag length testing. We implemented a data driven procedure for VAR lag length testing outlined in [30]. The tests were performed on daily-level data with a maximum lag length of 14 days (longer lags ensure stationarity). The results of the VAR lag testing, Table S1, include three measures: Akaike information criterion (AIC), Bayesian information criterion (BIC), and Hannan-Quinn criterion (HQ). The BIC values suggest a lag length of 3 or 4. We use a lag length of 3 because we anticipate quick responses by ISIS and governments. Results are substantively the same for a lag length of 4.

Lags	AIC	BIC	HQ
1	1.081	1.156	1.108
2	0.931	1.068	0.981
3	0.862	1.062	0.935
4	0.797	1.059	0.892
5	0.750	1.074	0.868
6	0.729	1.116	0.870
7	0.700	1.150	0.864
8	0.704	1.215	0.890
9	0.690	1.264	0.900
10	0.671	1.308	0.903
11	0.654	1.353	0.909
12	0.657	1.418	0.934

Table S1: VAR Lag Selection

Based on the VAR lag testing above, we use Granger causality as an initial diagnostic to assess correlations in the data and any causal interactions—i.e., within the narrow definition of "causality" in the Granger framework [31]. The Granger results presented here are simply a step within the framework of VAR modeling [32]. The results for all 20 Granger causality tests are shown in Table S2.

Granger models assess temporal relationships in a different way than CCF models. CCFs calculate the statistical significance of pairwise, dot-product correlations between specified lags and leads of two variables, whereas Granger models determine whether the estimated coefficients for a system of lags are jointly significantly different than zero. Consequently, the estimates generated by these models are not directly comparable. For example, the CCFs in Figure S1 include lags and leads over 20 time periods, while the Granger results are based on lags of three periods. That said, the Granger results are consistent with the CCF results. Of particular interest is the way ISIS responds to verbal conflict initiated by foreign governments, as shown in the first three rows of the results. Verbal conflict Granger-causes an increase in all three ISIS actions—attacks on civilians, attacks on militaries, and reciprocal threats—and has an especially statistically significant effect

on attacks on civilians. Material conflict initiated by foreign governments, by contrast, is weakly and insignificantly correlated with attacks on civilians (fifth row). The Granger results also show evidence of "reverse causality" in the two variables interest, such that *Attack Civilians* Granger-causes an increase in *Verbal Conflict*. As noted in the main paper, this result is consistent with the logic of strategic interaction between governments and extremists, and is thus unsurprising [33, 34, 35, 36]. Importantly, the Granger framework does not allow us to determine which direction of this relationship is stronger, or whether the apparent influence of one direction is spurious to the other [31, 37, 38]. Answering such questions requires more sophisticated methodologies, such as the the models discussed below.

	F-statistic	p-value
Verbal conflict -> Attack Civilians	22.92	0.00
Verbal conflict -> Attack Militaries	14.38	0.00
Verbal conflict -> Make Threats	14.43	0.00
Verbal conflict -> Material Conflict	14.49	0.00
Attack Civilians -> Attack Militaries	14.81	0.00
Attack Civilians -> Make Threats	20.43	0.00
Attack Civilians -> Material Conflict	11.11	0.00
Attack Civilians -> Verbal conflict	51.91	0.00
Attack Militaries -> Attack Civilians	12.10	0.00
Attack Militaries -> Make Threats	4.85	0.00
Attack Militaries -> Material Conflict	4.25	0.01
Attack Militaries -> Verbal conflict	10.57	0.00
Make Threats -> Attack Civilians	54.49	0.00
Make Threats -> Attack Militaries	8.45	0.00
Make Threats -> Material Conflict	2.08	0.10
Make Threats -> Verbal conflict	16.72	0.00
Material Conflict -> Attack Civilians	3.94	0.01
Material Conflict -> Attack Militaries	11.61	0.00
Material Conflict -> Make Threats	6.47	0.00
Material Conflict -> Verbal conflict	13.57	0.00

Table S2: Granger Causality

C Bayesian Structural VAR Model

C.1 Univariate vs Multivariate Models

Selecting an appropriate model that captures the causal processes driving strategic interactions is not a trivial matter. Such problems are widely discussed in the literature [39, 40, 41]. One option is univariate models. While both the CCFs and the Granger tests indicate the presence of a complex causal process, both are limited in various ways and do not capture an underlying causal structure. Univariate models do not reflect the dynamics and endogeneity in the data, or the multiple possible pathways of the causal mechanisms. A multivariate model is necessary to capture all these effects and tease out reciprocal behavior and endogeneity in the data [32, 39]. Such problems are not new in international relations; some examples of multivariate models can be found in the study of signaling between international actors [42] and reciprocal relations between global powers [3]. We show the advantages of a multivariate model in Figure S4 below and associated discussion on Gibbs sampling in Section C.5.

While we are particularly interested in how verbal attacks affected ISIS's treatment of civilians, governments and ISIS both have multiple possible behaviors at their disposal. And because these behaviors are interdependent, we require a more comprehensive model. Additionally, given the existing theoretical framework, the selected model should also allow for the inclusion of assumptions regarding these relationships. That is, what kind of responses should we expect to what kind of actions? The responses can occur both contemporaneously and over various lags, all of which should be modeled correctly. Thus, our selected model should allow for bidirectional effects, built-in theoretical assumptions, and both contemporaneous and lagged effects.

We implement a structural Bayesian time series approach [30, 39]. The model captures both contemporaneous and lagged relationships between the endogenous variables, which allows us to estimate bidirectional, short-term, and long-term effects. A single equation, containing the lagged and contemporaneous values of all variables in the system, is included for each endogenous variable. The endogenous variables are those defined in Section A above. The model further allows for structural identifications or specific causal expectations to be imposed on the dynamics of the system, which facilitates testing of different behavioral theories (built-in theoretical assumptions). For example, we might expect ISIS to respond to verbal conflict by attacking civilians, or we might instead expect governments to respond to ISIS attacks by engaging in material conflict.

The Bayesian structural VAR (B-SVAR) model has the following matrix form:

$$A_0 Y_t + \sum_{j=1}^{p} A_j Y_{t-j} = Z_t + \epsilon_t \qquad t = 1, ..., T. \tag{2}$$

The contemporaneous relationships among the variables, as determined by the underlying theory, are defined in 5×5 matrix A_0. We further discuss the specification of A_0 in Section C.5 below. The endogenous variables at time t are in the 5×1 vector Y_t, while A_j is a 5×5 matrix of the structural coefficients for the lagged endogenous variables Y_{t-j} at lag $t - j$. This approach enables the relationships between the endogenous variables (Y_t) to be included both in contemporaneous (A_0) and in lagged form (A_j). Z_t is a vector of the intercept, and ϵ_t is a 5×1 vector of normal i.i.d. structural shocks.

C.2 Causality Claims and Intervening Variables

Models that focus on endogenous relationships must address several issues. Intervening variables and variables that might have an effect on either variable, such as ISIS attacks against civilians, are one potential issue. Another is noncausality, as well as claims of causality versus timing. VAR models, and especially models that rely on impulse response analysis, such as our B-SVAR approach, interpret the relationships as causal given the structural reduced form error covariance identification—i.e., the contemporaneous identification in the A_0 matrix (Section C.5). The model does not assume any exogeneity restrictions (the requirement for weak exogenous effects of one endogenous variable on another), associated with noncausality, before estimation [39, 43].

Any causal claims also need to be understood within the modeling framework. The focus here is the timing and size of the responses between the variables of interest. Our approach allows us to study these responses over the entire period or within any specific periods, not between specific instances of threats and attacks, for example. While we cannot make a claim that a specific threat against ISIS *"caused"* a specific attack against civilians, we can study and forecast the temporal relationship between these events.

While intervening variables might exist, assuming serially uncorrelated residuals, in reduced form VAR models the shocks are exogenous to the variables as they are unpredictable random errors [30, pp. 56-58]. For both substantive and methodological reasons, we do not include control variables in the main model [42]. In Section F below, we show results for models that incorporate control variables.

C.3 Why use a Bayesian model

The preliminary analysis shows that there are multiple causal paths and dynamics that exist in the data, which need to be fully modeled. Additionally, the structural identification of the causal mechanisms is essential when applying a theoretical framework to the strategic interactions. The structural equations enable the addition of predetermined behavioral expectations based on existing theories that can then be tested directly against one another to select the best fit for the data. Thus, possible relations are identified both in terms of their short-term and long-term dynamics.

Given the complexity identified by the initial models, the B-SVAR model addresses issues of endogeneity and specification uncertainty. The model does not simplify the dynamics in order to model them; rather, it retains the identified complexity [42]. This is enabled by the inclusion of the full dynamics of the lagged relationships dependent on the contemporaneous causal relationships. The model contains a large number of parameters that need to be developed correctly—structural identifications, numerous priors, etc. We do this for the main period of interest, the three separate periods we study, for each ICEWS event code of interest, for filtered and unfiltered data, and then for ISIS, Al-Qaeda in Iraq, and Boko Haram. This breadth creates significant problems of scale, which we discuss in detail below. Our multiple-equation model enables us to consider each of these parameters for each subset of the data. Previous studies of terrorism and government actions have not fully accounted for this complexity and model scale.

The Bayesian nature of the model is crucial in disentangling the interactions. Priors have been chosen based on knowledge of the data and careful testing for fit. The priors are essential in building a model with a good fit and improved accuracy. The various priors, including the structural

identification (acting as a prior) allow for a more transparent modeling as they show clearly what are the theoretical and modeling frameworks that are part of the model.

C.4 Choice of Priors

The priors used in the model represent a set (or sets) of beliefs regarding the model coefficients and structure of the system. These beliefs/priors are included in the model by correlating them across equations [44]. For the final version of the models, we use a modified version of the Sims and Zha prior, which puts low probability on nonzero values for the coefficients at the most distant lags [45]. Before selecting priors for the model, a prior specification search is necessary to assess which priors are the best fit for the data. We included 160 combinations of various priors and estimated the posterior and in-sample fit measures for a reduced form VAR model.

The included priors are based on the framework discussed in the R MSBVAR package (list and descriptions below quoted from package documentation) [46]:

- lambda0: [0; 1], Overall tightness of the prior (discounting of prior scale).
- lambda1: [0; 1], Standard deviation or tightness of the prior around the AR(1) parameters.
- lambda3: Lag decay (¿ 0, with 1=harmonic)
- lambda4: Standard deviation or tightness around the intercept ¿ 0
- lambda5: Standard deviation or tightness around the exogneous variable coefficients ¿ 0
- mu5: Sum of coefficients prior weight ≥ 0. Larger values imply difference stationarity.
- mu6: Dummy initial observations or drift prior ≥ 0. Larger values allow for common trends.
- nu: Prior degrees of freedom, m + 1
- qm: Frequency of the data for lag decay equivalence. Default is 4, and a value of 12 will match the lag decay of monthly to quarterly data. Other values have the same effect as ”4”
- prior: One of three values: 0 = Normal-Wishart prior, 1 = Normal-flat prior, 2 = flat-flat prior (i.e., akin to MLE)

The priors that we included in the testing were:

- lambda0: 0.7, 0.8, 0.9, 1
- lambda1: 0.1, 0.2, 0.3, 0.4, 0.5, 0.7, 0.8, 0.9, 1
- lambda3: 0.5, 1, 1.5, 2, 2.5
- lambda4: 0.3
- lambda5: 0.1
- mu5: 0
- mu6: 0, 2
- nu: degrees of freedom
- qm: 4

- prior: Normal-Wishart prior

Figures S2 and S3 show the results of the prior specification searches. The selection of the most appropriate priors is based on the root mean squared errors and the marginal posteriors of the data. Prior evaluation searches set one (Figure S2) include lambda1 values 0.1, 0.2, 0.3, 0.4, 0.5 (first columns present the first lambda1 value, second columns present the second lambda1 value, etc., shown in orange lines across lambda1 labels); and lambda3 values 1, 1.5, 2, 2.5 (bottom rows present the first lambda3 value, second rows present the second lambda3 value, etc., shown in green lines across lambda3 labels).

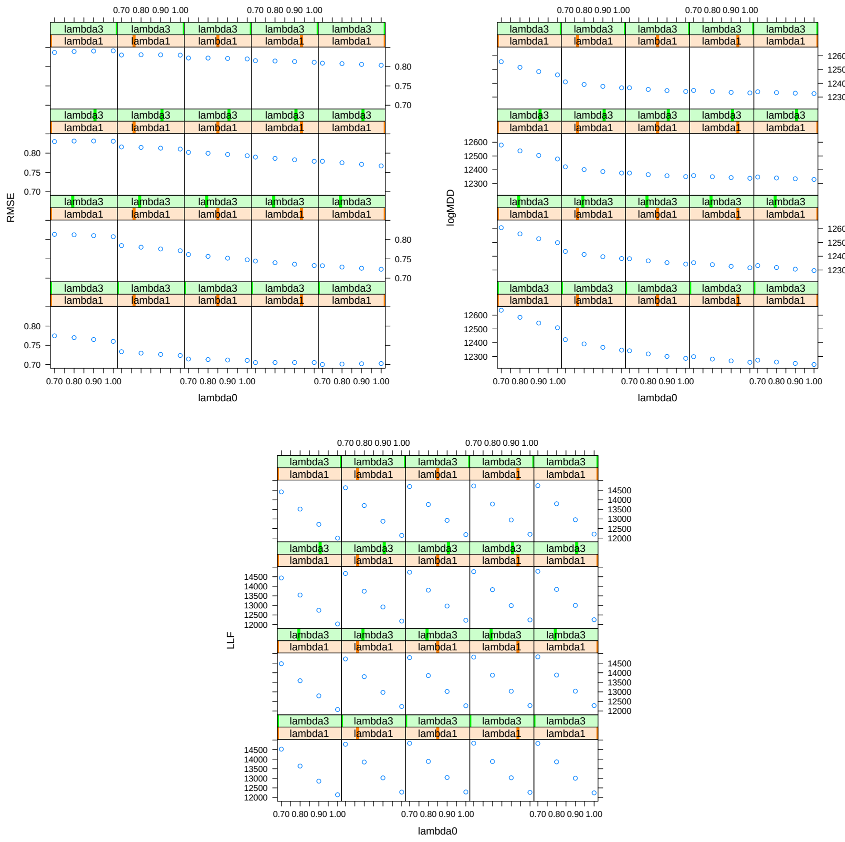

Figure S2: Prior Evaluation Searches Set One. Evaluation based on root mean squared error (RMSE) on the left, log marginal data density (logMDD) in the middle, and log-likelihood (LLF) on the right. Each circle represents a particular combination between lambda0 (values on x-axes), lambda1 (values in orange), and lambda3 (values in green). Resulting metrics are on the y-axes.

Prior evaluation searches set two (Figure S3) include lambda1 values 0.5, 0.7, 0.8, 0.9, 1 (first columns present the first lambda1 value, second columns present the second lambda1 value, etc., shown in orange lines across lambda1 labels); and lambda3 values 0.5, 1, 1.5, 2 (bottom rows present the first lambda3 value, second rows present the second lambda3 value, etc., shown in green lines across lambda3 labels). Both sets of prior evaluation searches include the full set of lambda0 values listed above and shown on the x-axes in the figures.

Based on the prior specification searches, the final model includes the following priors: lambda0=0.8, lambda1=0.9, lambda3=1, lambda4=0.3, lambda5=0.1, mu5=0, mu6=2, as well as the structural

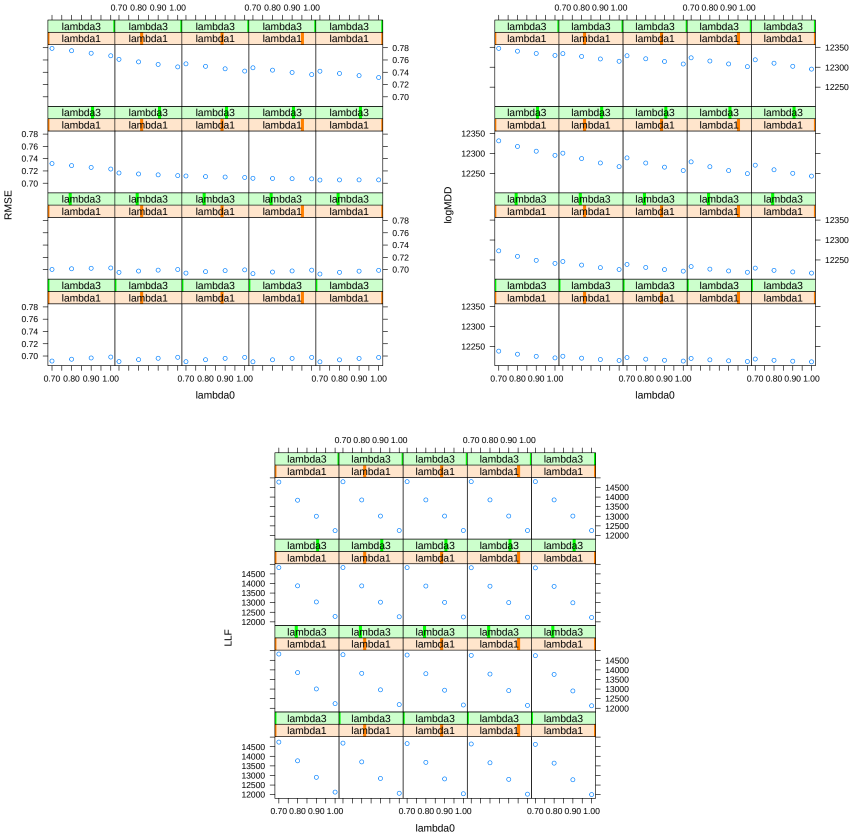

Figure S3: Prior Evaluation Search Set Two. Evaluation based on root mean squared error (RMSE) on the left, log marginal data density (logMDD) in the middle, and log-likelihood (LLF) on the right. Each circle represents a particular combination between lambda0 (values on x-axes), lambda1 (values in orange), and lambda3 (values in green). Resulting metrics are on the y-axes.

identification discussed below. The selected priors that are used in the final model are tight priors around the AR(1) parameters, the intercept, and the exogenous variable coefficients, with a harmonic decay and a lag length of 8 (based on the lag testing discussed above).

C.5 Structural Identification

As discussed in the sections above, the model allows for structural expectations to be imposed on the dynamics of the system, which facilitates testing of specific behavioral theories (built-in theoretical assumptions). For example, we might expect ISIS to respond to verbal conflict by attacking civilians, or we might instead expect governments to respond to ISIS attacks by engaging in material conflict. These contemporaneous relationships among the variables, as determined by the underlying theory or expectations, are defined in 5×5 matrix A_0. The addition of the structural identification of the contemporaneous causal relationships does not limit the interactions that occur with a delay (captured by A_j). The structural identifications can be evaluated (comparing the fit of the different possibilities), which provides evidence for the direction of the interactions and the underlying causal mechanisms. The most appropriate specification should be based on such evaluation.

The structural identification in structural VAR models relates to claims about contemporaneous relationships, or the immediate (within the same day) effects of shocks in one variable as they enter the system of equations. This also relates to the speed of the response of the variables. Within the SVAR framework, shocks are defined as unexpected changes (specified as positive or negative) in one of the variables that can be traced as they enter the system. Contemporaneous refers to the level of measurement allowed by the data—daily, in our case. The model does not make assumptions about "simultaneity;" that is, it does not claim that such relationships occur concurrently, but that they could occur within the same day.

The structural identification allows for expectations and theories to be represented as competing sets of possible relationships/interactions or behavioral equations (matrix A_0). We focus on the responses of ISIS to actions initiated by foreign governments. We include a control model, "No Response," which assumes no contemporaneous relationships between ISIS actions and government actions. The theoretical basis for this model is that the actions of both ISIS and foreign governments may be motivated by unrelated concerns, such as domestic politics, in which case neither actor pays immediate attention to, or is contemporaneously influenced by, the behavior of its adversary.

A second model, "Deterrence," assumes that ISIS responds to material attacks by attacking both civilians and militaries. This model is consistent with the traditional deterrence logic of conflict [47], as well as "costly signaling" approaches [48, 49], which assume that confrontations follow an escalatory process, where actors engage in increasingly hostile actions in order to deter their adversaries from escalating further. In this specification, cheap talk is inconsequential; governments and terrorists respond contemporaneously only to material conflict [50].

The third model, "Reciprocity," builds upon the Deterrence model but adds the assumption that ISIS responds in kind to government actions. That is, ISIS responds to material attacks by engaging in attacks of its own (either against militaries or civilians), and it responds to verbal attacks by making reciprocal threats. This model incorporates long-standing theoretical arguments on the role of reciprocity in international relations [51, 52, 53, 54].

The "Credibility" model adds behavioral complexity to the previous models. It specifies that when targeted with verbal attacks, ISIS responds with threats of its own (as in the Reciprocity model), and that when targeted with material attacks, it responds with attacks against militaries and/or civilians (as in the Deterrence model). However, it also specifies that ISIS responds to verbal conflict by attacking civilians. As discussed in the main paper, the theoretical basis for this assumption is that threats, denunciations, rejections, and other forms of verbal conflict undermine ISIS's second-order beliefs about its credibility, thus creating incentives for ISIS to engage in increasingly extreme acts of violence and credibly signal its strength and resolve. While the No Response model assumes no contemporaneous responses, and the Deterrence and Reciprocity models assume interactions that reflect traditional theoretical expectations, the Credibility model theorizes terrorists as aware of their reputations, sensitive to actions and statements by foreign governments that call those reputations into question, and eager to find opportunities to violently exhibit their credibility.

We also estimate two models that use an alternative direction for the structural specification. These models directly test the hypothesis that the contemporaneous responses are primarily on the government side—for example, when an ISIS attack against civilians is followed by a condemnation by governments. The "Government" model specifies contemporaneous responses only on the side of governments. Specifically, governments respond to any ISIS action with verbal conflict, and they respond to ISIS material actions with material conflict. In this model, ISIS is not immediately sensitive to government actions. The "Government Plus" model builds on the same assumptions but

allows ISIS to contemporaneously respond to material actions by governments with material actions of its own. This model proposes that governments respond contemporaneously to all ISIS actions, but ISIS only responds contemporaneously to material government actions (*Material Conflict*) and is insensitive to verbal attacks. Because the structural identification for this model omits the *Verbal Conflict* → *Attack Civilians* causal direction and instead prioritizes government responses, we can compare the fit of this model to the Credibility model and directly test the expectation that verbal attacks by governments causally lead to material responses by ISIS. Further, because the Government Plus model also includes the most "free" parameters (two more than the Credibility model), we can also test potential model overfitting (i.e., inflated fit statistics due to a high number of estimated parameters).

The structural identifications (and associated A_0 matrices) are shown in Table S3. Each block is a unique structural model, and each row is a single equation in the corresponding model. The columns are the contemporaneous shocks (unexpected changes) in an endogenous variable that enter each equation. An "X" in a cell represents a "free" parameter to be estimated in the model. These are hypothesized contemporaneous effects from a column variable affecting the row equation. An empty cell indicates no hypothesized contemporaneous relationship. The diagonals of each model block contain an "X" for each variable because a contemporaneous change (shock) in a variable should lead to a response in the same variable.

We compare the six models depicted in Table S3—where the models are identical besides the structural identifications—using the fitted B-SVAR posterior probabilities [42]. Table S4 compares the results using the sum of the log probability values (LLF) and the log posterior marginal data densities (LMDD) of the six tested models. The best-fitting model is Credibility with an LLF of -5796.06, a noteworthy difference of 26.99 compared to the Reciprocity model. The LLF of the Credibility model is also significantly larger (a difference of 9.47) than the Government model, showing evidence that the interactions are notably stronger in the responses by ISIS. The Credibility model also outperforms the Government Plus model on LLF despite the smaller number of "free" parameters estimated. Although the Government Plus model performs slightly better than the Credibility model on LMDD, the difference is small (2.54) and, in light of the LLF result, does not obviously indicate that Government Plus is the superior model. These results show that the omission of the *Verbal Conflict* → *Attack Civilians* effect is incorrect, and that ISIS responses to government-initiated verbal attacks should be included for a better model fit. By contrast, the inclusion of contemporaneous government responses in the structural identification only marginally improves fit. This result supports the Credibility model over alternative specifications [55]. Due to the support for the Credibility model, we selected its contemporaneous specification as the structural identification (matrix A_0 in Eq. 2) in the B-SVAR model in the main paper, as well as for all tests below. Unless otherwise indicated, any below reference to "the model" refers to the Credibility model.

To evaluate the fit of multivariate models versus univariate models, as discussed in Appendix C.1, we test whether the multivariate model used here has an advantage over univariate models that do not take into account the structural identification discussed above. Gibbs sampling is used as a method for statistical inference and, in the case of B-SVAR models, depicts whether the structural identification is correct, or showing effects different than zero [56, 57]. We analyze the output from the Gibbs sampler for the Credibility B-SVAR model in Figure S4. All results and tests below use a burnin of 50,000 iterations and 100,000 posterior draws.

The nonzero effects captured by the structural multivariate model shown in Figure S4 were not

Table S3: Contemporaneous Relationships for Structural Models

Model block	Variable	*Verbal Conflict*	*Material Conflict*	*Attack Civilians*	*Attack Militaries*	*Make Threats*
No Response	*Verbal Conflict* (Govmt.)	X				
	Material Conflict (Govmt.)		X			
	Attack Civilians (ISIS)			X		
	Attack Militaries (ISIS)				X	
	Make Threats (ISIS)					X
Deterrence	*Verbal Conflict* (Govmt.)	X				
	Material Conflict (Govmt.)		X			
	Attack Civilians (ISIS)		X	X		
	Attack Militaries (ISIS)		X		X	
	Make Threats (ISIS)					X
Reciprocity	*Verbal Conflict* (Govmt.)	X				
	Material Conflict (Govmt.)		X			
	Attack Civilians (ISIS)		X	X		
	Attack Militaries (ISIS)		X		X	
	Make Threats (ISIS)	X				X
Credibility	*Verbal Conflict* (Govmt.)	X				
	Material Conflict (Govmt.)		X			
	Attack Civilians (ISIS)	X	X	X		
	Attack Militaries (ISIS)		X		X	
	Make Threats (ISIS)	X	X			X
Alternate direction						
Government	*Verbal Conflict* (Govmt.)	X		X	X	X
	Material Conflict (Govmt.)		X	X	X	
	Attack Civilians (ISIS)			X		
	Attack Militaries (ISIS)				X	
	Make Threats (ISIS)					X
Govmt. Plus	*Verbal Conflict* (Govmt.)	X		X	X	X
	Material Conflict (Govmt.)		X	X	X	
	Attack Civilians (ISIS)		X	X		
	Attack Militaries (ISIS)		X		X	
	Make Threats (ISIS)					X

depicted by the univariate models discussed in Appendix B. The inclusion and correct identification of the underlying structural mechanisms is essential for a good model fit. Univariate models do not capture the endogeneity in the data, or the multiple possible pathways of the causal mechanisms that can be traced in multivariate models, as shown in Figure S4. Multivariate time series methods and VAR models have been shown to allow for the tracing of multiple causal paths in the data [43, 58], which is essential in answering questions similar to the one posed here. Structural VAR models are especially appropriate in characterizing the effects of endogeneity [43], and the results from the Gibbs Sampling in Figure S4 show that the contemporaneous identification that we selected above should indeed be taken into account to capture the interactions in their entirety.

Table S4: Posterior Model Summaries. LLF = the sum of the log probability values; LMDD = log marginal data density.

Model	*LLF*	*LMDD*
No Response	-5926.81	-6578.29
Deterrence	-5839.39	-6498.26
Reciprocity	-5823.05	-6485.48
Credibility	-5796.06	-6466.26
Alternate direction		
Government	-5808.53	-6478.31
Govmt. Plus	-5800.53	-6463.72

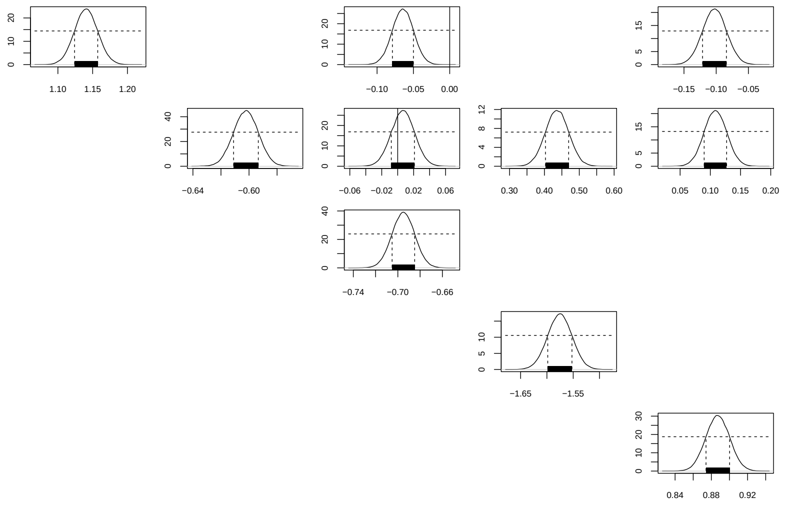

Figure S4: Gibbs Sampling, parameter density summary for the Credibility B-SVAR model. Burnin of 50,000 iterations and 100,000 posterior draws.

D Discussion of Final Results

Impulse response functions (IRFs) allow for the tracing of the responses of a system of equations to shocks in selected variables, depicting the dynamics between endogenous variables in VAR and B-SVAR models. They present graphically the effects of shocks in endogenous variables in a system as they enter specific equations, allowing for the responses to these shocks to be traced over specified periods [39, 44]. For reference, Figure S5 visualizes the impulse response analysis of the unrestricted "No Response" model. The impulse response analysis of the "Credibility" model, with the structural identification and priors discussed above, is visualized in the IRFs in Figure S6. The similarity of the IRFs between the two specifications, particularly for the variables of interest, confirm that the main finding of interest is not an artifact of the structural identification.

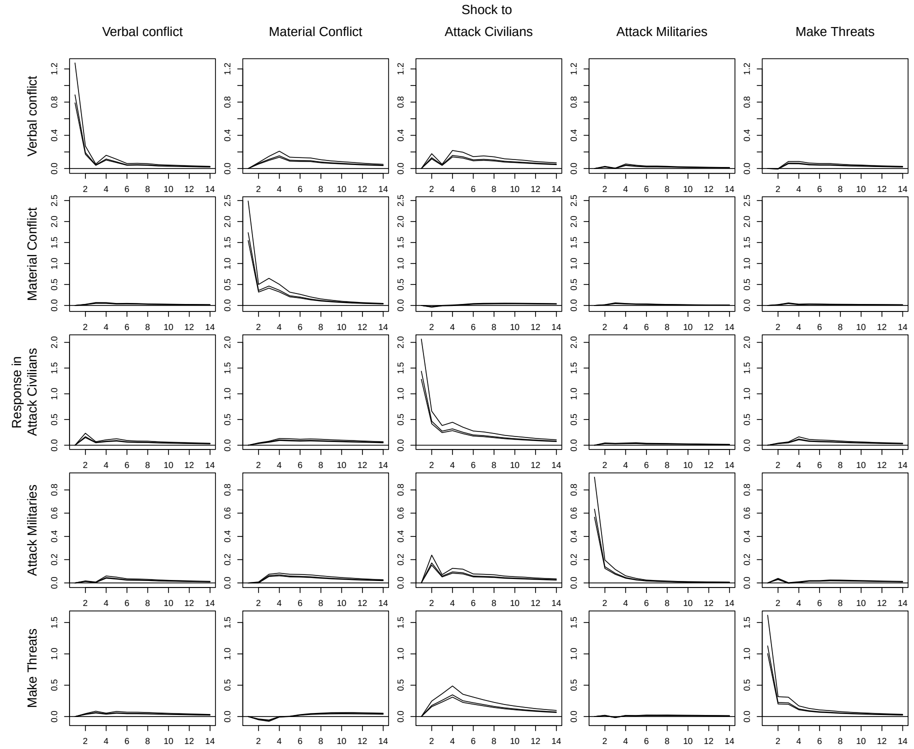

Figure S5: Impulse response functions for the "No Response" B-SVAR model using unfiltered ICEWS data with Monte Carlo integration. All shocks are positive. The error bands are 99% posterior pointwise credible intervals around the median estimates, computed using eigenvector decomposition of the full stacked responses. Based on Gibbs Sampling with a burnin of 50,000 iterations and 100,000 posterior draws, and 5,000 final posterior draws for the MSBVAR posterior forecast density. Responses are over a fourteen day period and are based on one standard deviation shocks.

The results are based on a posterior of IRFs by Monte Carlo integration following an estimation of the posterior mode for the B-SVAR model using the priors and structural identification discussed above (full discussion of the procedure in [45] and [42]). Posterior sample objects are generated by the Gibbs Sampling procedure discussed above, with a burnin of 50,000 iterations and 100,000 posterior draws, and normalization as suggested by [59] and [60]. All impulse responses are for positive one standard deviation shocks to the system of equations, chosen for easier interpretation and uniformity across the comparisons. The IRFs include 5,000 final posterior draws (from the MCMC sampling) for the MSBVAR posterior forecast density. The error bands for the impulse

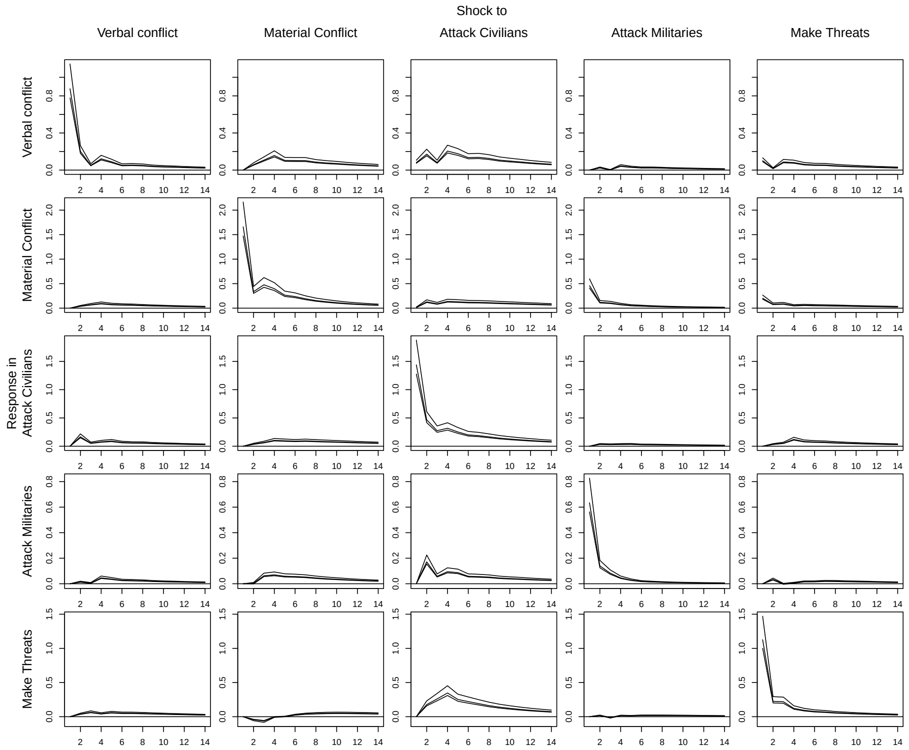

Figure S6: Impulse response functions for the "Credibility" B-SVAR model using unfiltered ICEWS data with Monte Carlo integration. All shocks are positive. The error bands are 99% posterior pointwise credible intervals around the median estimates, computed using eigenvector decomposition of the full stacked responses. Based on Gibbs Sampling with a burnin of 50,000 iterations and 100,000 posterior draws, and 5,000 final posterior draws for the MSBVAR posterior forecast density. Responses are over a fourteen day period and are based on one standard deviation shocks. Panels of interest are outlined in red.

responses are computed using eigenvector decomposition of the full stacked responses over fourteen days [44]. They are 99% posterior pointwise credible intervals around the median estimates. The error bands of 99% that we employ far exceed the 68% (approximately one standard deviation) recommended by [56], which speaks to the excellent model fit that we have achieved.

The findings in the main text of the paper (Figure 3 of main paper), are derived from columns 1–2, rows 3–5 of Figure S6, outlined in red. These results are based on one-standard-deviation shocks, which we then convert to numeric values—i.e., number of attack events as a response to number of verbal/material conflict events. We then compute the peak effect or largest response. The peak effect, which we show in Table 1 in the main text, observed at the two-day mark, translates to one additional attack on civilians for a shock equivalent to 3.15 verbal attacks by foreign governments.

As discussed in Section A above, we tested both filtered and unfiltered data. The unfiltered data err on the side of including all events, even if some of those events are duplicates, while the filtered data err on the side of excluding duplicates, even if some true events are also excluded. The main results use the unfiltered data (Figure S6). We ran the same tests and procedures on the filtered data to assess whether our findings are sensitive to filtering. The IRF results with the filtered data are in Figure S7. They are nearly identical to the main results. The findings in the main paper hold regardless of filtering procedure.

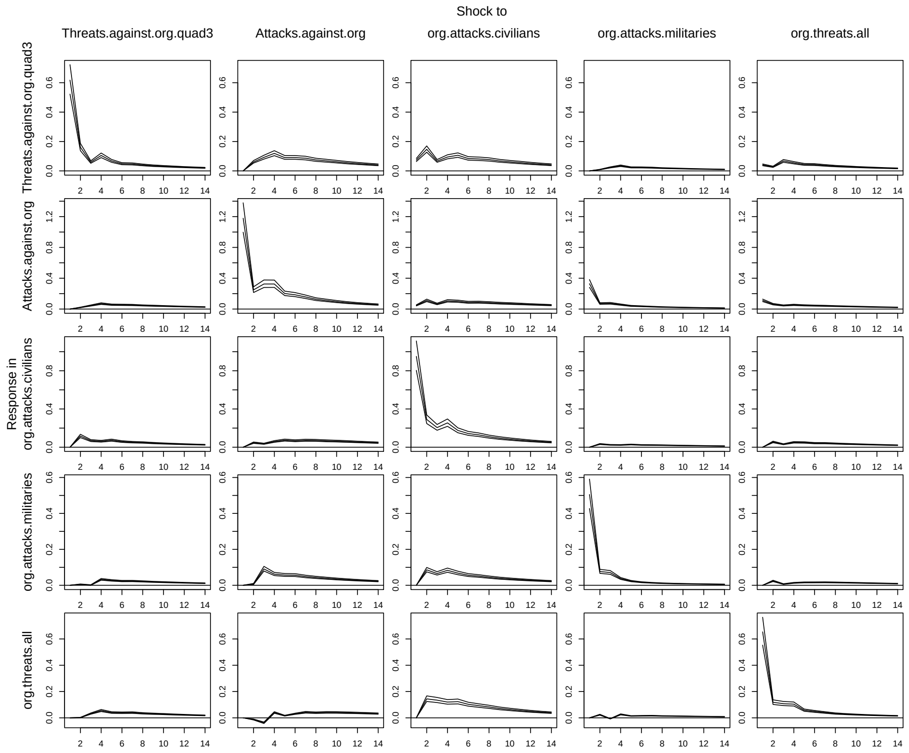

Figure S7: Impulse response functions for the B-SVAR model using **filtered ICEWS data** with Monte Carlo integration. All shocks are positive. The error bands are 99% posterior pointwise credible intervals around the median estimates, computed using eigenvector decomposition of the full stacked responses. Based on Gibbs Sampling with a burnin of 50,000 iterations and 100,000 posterior draws, and 5,000 final posterior draws for the MSBVAR posterior forecast density. Responses are over a fourteen day period and are based on one standard deviation shocks. Quadrant of interest is outlined in red.

E Convergence Diagnostics

A crucial aspect of Bayesian models is their convergence, or whether the sampler is adequately approximating the specified posterior distribution. Markov chain Monte Carlo (MCMC) based methods, such as those used here, draw samples from a posterior distribution, which is assumed to have converged to an equilibrium. If there is no convergence, the reported results are based on flawed assumptions. As such, convergence diagnostics are crucial when reporting results from such models. To assess the results from our B-SVAR model, we run several convergence diagnostics.

Upon reviewing the summary of the free parameters in the MCMC Gibbs Sample object, we inspect the traceplots in Figure S8. The plots do not show any issues such as multimodality or wide posterior tails. The sampler has reached normal distributions for all chains. The traceplots for all chains indicate convergence.

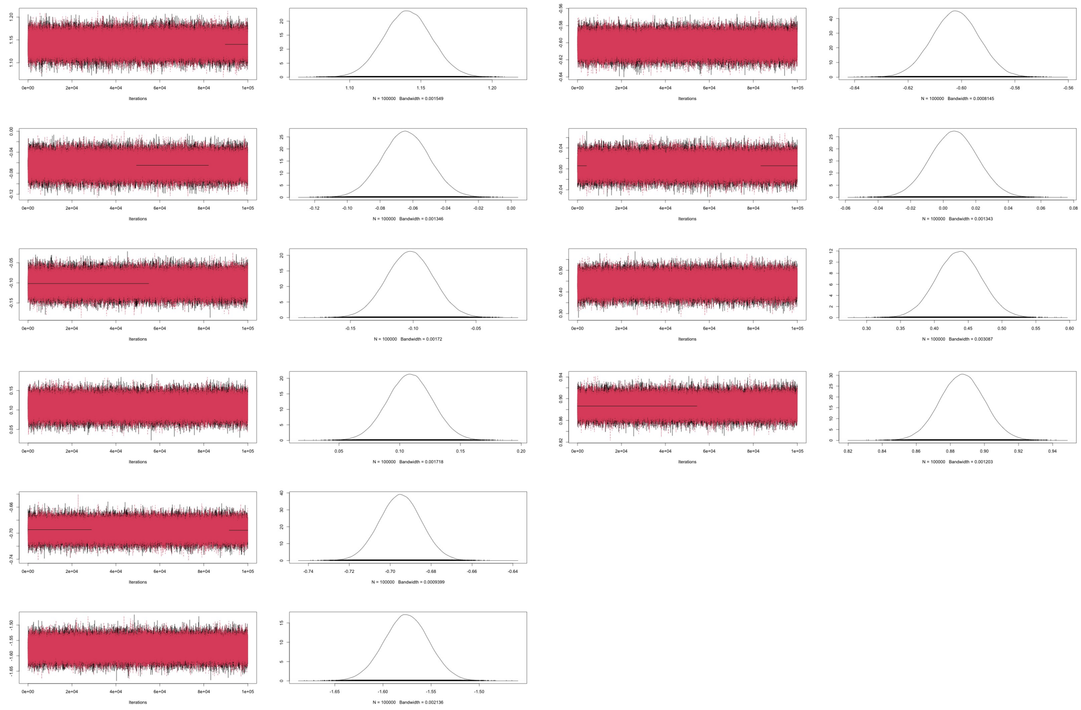

Figure S8: Traceplots for Chains.

Next, we test for autocorrelation in the chains. The results for all chains (two clusters ran in parallel) are in Figure S9 and they do not show any problems with autocorrelation.

We checked for possible cross correlations, and found no indication of such issues. We also ran the Raftery and Lewis diagnostic, which produced very low dependence factors (close to one) indicating very quick convergence [61], and we ran the Heidelberg and Welch diagnostic with passing results for all stationarity and halfwidth tests [62].

We follow with Gelman-Rubin diagnostics in Figure S10, which show relatively quick convergence for all chains [63, 64]. All convergence diagnostics indicate that the B-SVAR model has converged.

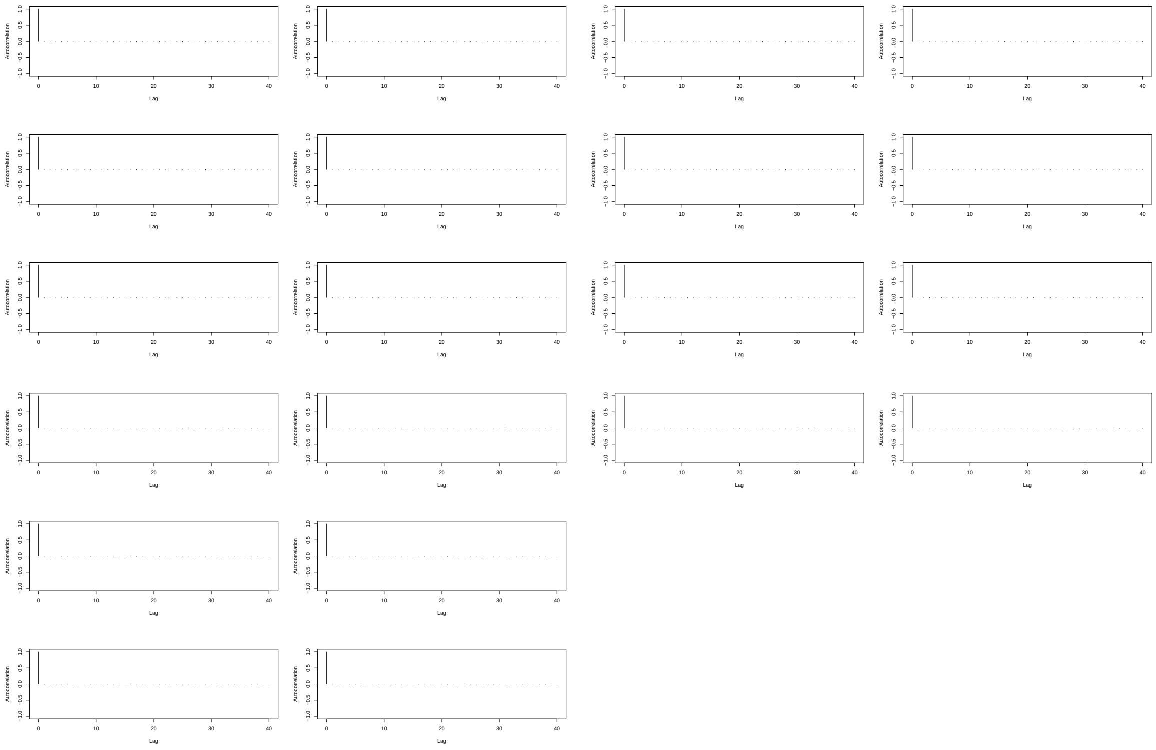

Figure S9: Tests for Autocorrelation in the Chains.

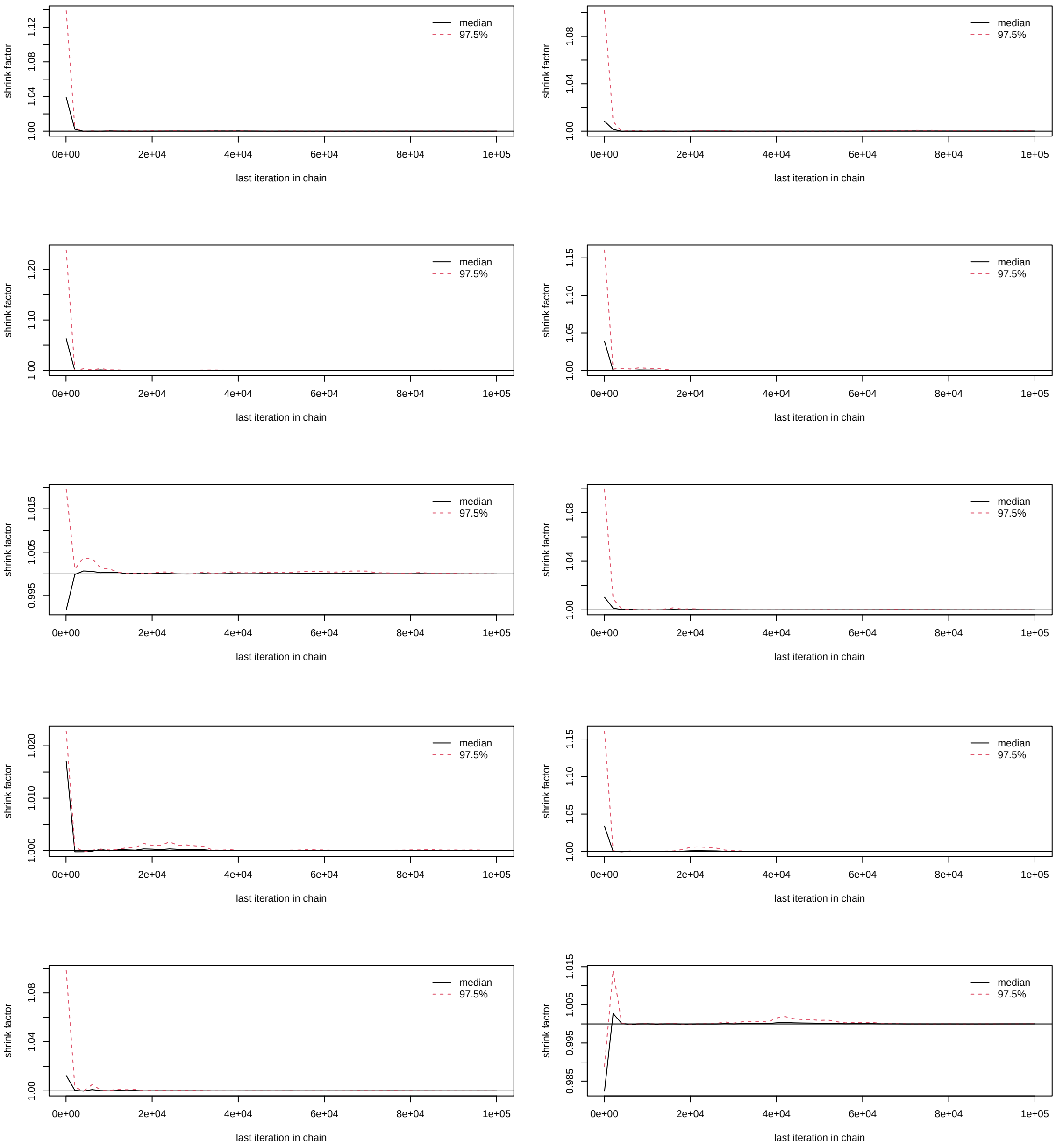

Figure S10: Gelman-Rubin Convergence Diagnostics.

F Additional Models and Robustness Checks

The main analysis casts a wide net, covering all varieties of interaction among ISIS, civilians, and governments, and with a model specification that incorporates into the system of equations only those variables that we expect to be causally related at the daily level. Here, we explore subsets of the data and additional model specifications in order to assess the proposed credibility mechanism and the overall robustness of the results.

F.1 Analysis of data subsets

Our results may be driven by, or limited to, only specific types of interactions. Accordingly, we re-estimate the main model on subsets of the ICEWS data. Figure S11 shows the estimated IRFs for the effect of *Verbal Conflict* on *Attack Civilians* across paired subsets of data.

First, we separate interactions between ISIS and western governments from interactions between ISIS and non-western governments, where "western" is defined as any country with a Correlates of War country code less than 400.[1] This specification assesses whether ISIS's responsiveness to verbal attacks is confounded by a Huntingtonian "clash of civilizations" or "west versus the rest" dynamic rather than resulting from the hypothesized credibility deficit [65]. The results, summarized in Figure S11(a), show that the effect of verbal conflict is substantively similar across subsets of data, which contradicts the possibility of a civilization-based clash.

Second, we separate verbal attacks initiated by countries that were active participants in the multilateral military coalition against ISIS from verbal attacks sent by other governments. For this analysis, we consider any government that was directly involved in military actions against ISIS or provided military aid for those actions as a member of the global anti-ISIS coalition.[2] Drawing on the logic of credibility deficits, we anticipate that ISIS responded more strongly to members of the anti-ISIS coalition than to non-members. When extremists evaluate their reputations and assess the impact of their efforts at costly signaling, they look to the responses of the actors toward whom their actions are most clearly directed—in this case, those governments that had ISIS in their sights. As Figure S11(b) shows, this expectation is borne out by the data, further substantiating the credibility mechanism.

Third, we distinguish between ISIS attacks that occurred in Iraqi and Syrian provinces where ISIS controlled territory, and ISIS attacks that occurred outside of ISIS-controlled provinces. These models assess the possibility that ISIS's apparent responsiveness to verbal conflict was in fact driven by a need to police its claimed territory and exert control over local populations. The fluid nature of ISIS's borders, combined with the imprecision of geolocated event data, pose a methodological challenge. By focusing on the province level, we are able to highlight those attacks that were most likely to involve territorial concerns, whether those attacks occurred within areas ISIS explicitly controlled, along the borders of those areas, or in nearby areas that were at high risk

[1] This criterion defines "western" as governments in the Americas or Europe. See `https://correlatesofwar.org/data-sets/cow-country-codes-2/`.

[2] The specific list of countries is Albania, Australia, Belgium, Bosnia and Herzegovina, Bulgaria, China, Croatia, Czech Republic, Estonia, Greece, Hungary, Indonesia, Kuwait, Lebanon, Slovenia, Spain, Afghanistan, Cameroon, Canada, Chad, Denmark, Egypt, France, Germany, Iran, Iraq, Italy, Jordan, Libya, Morocco, Netherlands, Niger, Nigeria, North Macedonia, Poland, Portugal, Qatar, Russia, Saudi Arabia, Singapore, Sweden, Syria, Turkey, United Arab Emirates, United Kingdom, and United States.

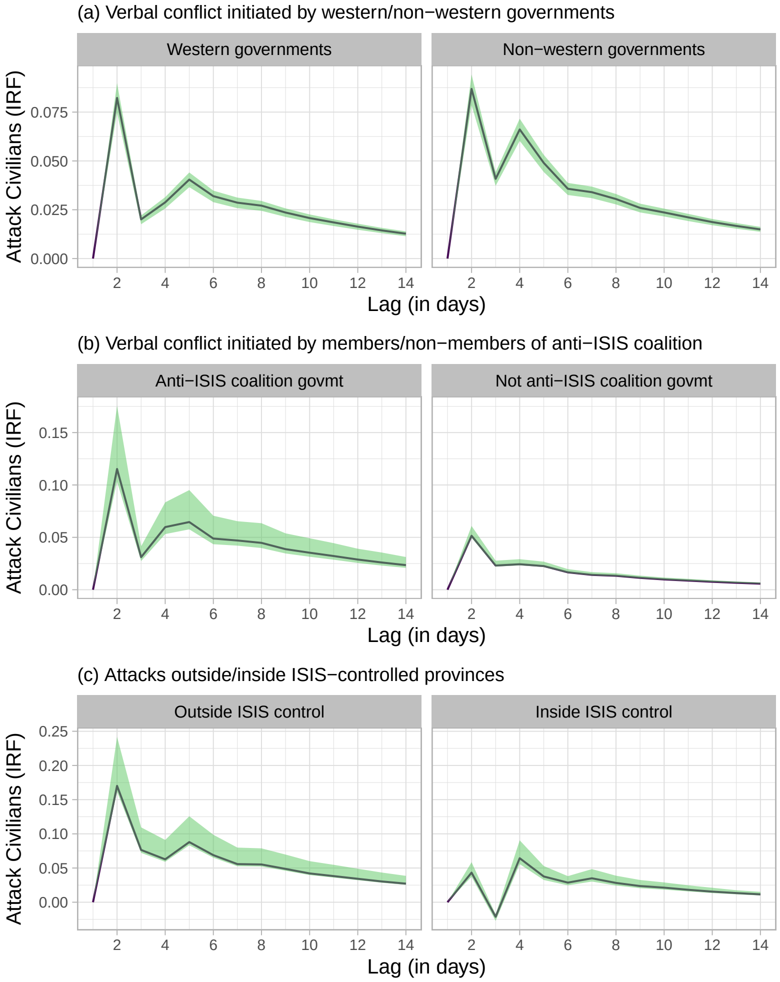

Figure S11: Impulse response functions for the B-SVAR model using subsets of ICEWS data. Panels illustrate effect of *Verbal Conflict* (column) on *Attack Civilians* (row) for each specified subset. In (a), effect of verbal conflict initiated by western governments versus non-western governments. In (b) effect of verbal conflict initiated by governments that participated in the global anti-ISIS military coalition versus verbal conflict initiated by governments that did not participate. In (c) effect of verbal conflict on ISIS attacks in provinces it controlled versus outside areas.

of ISIS expansion.[3] Figure S11(c) shows the results. We note in particular that (1) ISIS's attacks on civilians outside of provinces it controlled were in fact *more* responsive to verbal conflict, contrary to the possibility that ISIS attacks were driven purely by territorial concerns, and (2) even within provinces it controlled, ISIS was still significantly responsive to verbal conflict, though at a lower magnitude. Overall, these results show that although ISIS used attacks on civilians to accomplish numerous goals (territorial control among them), it was also motivated in part by the strategic incentive to close the credibility deficit generated by government-initiated verbal attacks.

[3] In Syria, the provinces are Ar Raqqah, Aleppo, and Dayr az Zawr. In Iraq, the provinces are Al Anbar, Nineveh, Kirkuk, and Salah ad Din. In both cases, we defined events as occurring within ISIS-controlled provinces only during time periods in which ISIS controlled major cities within those provinces.

F.2 Analysis with exogenous controls

As an additional robustness check, we estimated a model that incorporates exogenous covariates. As discussed in the main paper and elsewhere in this appendix, given the high-resolution nature of the data, combined with the B-SVAR model's focus on how shocks affect the overall system of equations, exogenous covariates are unlikely to significantly impact our main results. Nonetheless, to confirm that this is indeed the case, we added four additional variables to the model: the geographic extent of ISIS's occupied territory; the size of ISIS's primary contingent in Iraq and Syria, defined as number of known ISIS fighters; ISIS's economic capacity, defined as known ISIS revenues in millions of US dollars; and time periods immediately preceding substantial gains or losses in territory, which are most likely to involve follow-up attacks and/or multiday campaigns. We derived these measures from reports published by (1) the US Department of State's Bureau of Counterterrorism's *Country Reports on Terrorism*;[4] (2) the *Mapping Militants* project at Stanford's Center for International Security and Cooperation;[5] (3) the Rand Corporation;[6] and (4) the US Treasury Department. Figure S12 illustrates the main set of relationships once we control for these factors. As expected, the results are virtually unchanged from the main analysis.

F.3 Analysis of ISIS-inflicted fatalities

Throughout the paper, we focus generically on ISIS attacks on civilians, which include a range of actions, from armed assaults and executions to abductions and sexual assault. Such attacks often involved civilian fatalities, but not as a matter of course. Further, ISIS often deliberately turned to barbaric tactics like public humiliation and enslavement that conveyed a willingness to inflict harm on civilians but did not necessarily lead to large numbers of deaths. Restricting the analysis to only consider civilian deaths thus omits the many possible actions that are included in the broader *Attack Civilians* variable, and which ISIS utilized in establishing its reputation. Nevertheless, as a final robustness check, we replace the measure of ISIS attacks on civilians with a daily count of fatalities associated with those attacks [13]. The results in Figure S13 match the general finding from the main paper: ISIS responds to government-initiated verbal conflict by killing more civilians. The dynamics of these interactions are largely identical to the results focusing on the broader *Attack Civilians* variable.

[4] `https://www.state.gov/country-reports-on-terrorism-2/`

[5] `https://cisac.fsi.stanford.edu/mappingmilitants/profiles/islamic-state`

[6] `https://www.rand.org/topics/the-islamic-state-terrorist-organization.html`

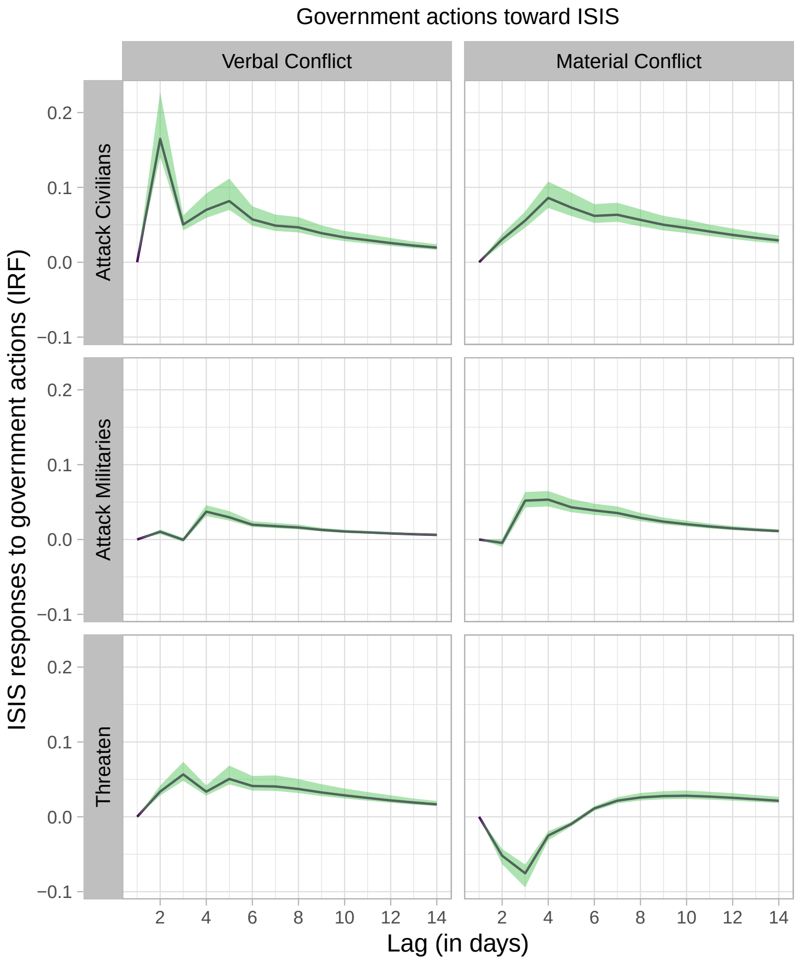

Figure S12: Impulse response functions for the B-SVAR model, with exogenous controls included for ISIS territorial control, number of fighters, revenues, and multiday campaigns.

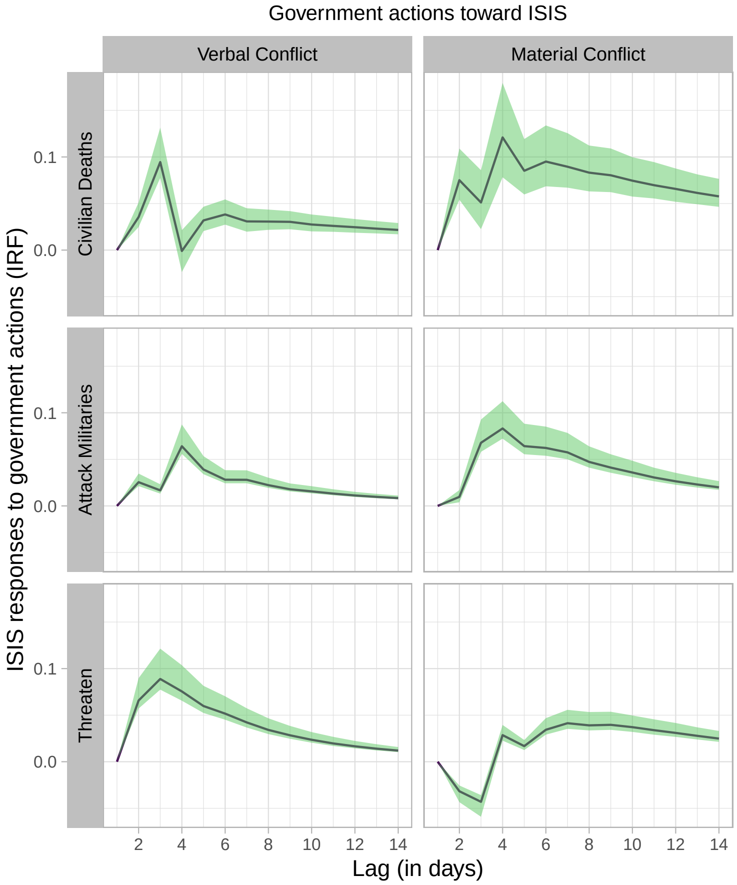

Figure S13: Impulse response functions for the B-SVAR model substituting attacks against civilians with civilian fatalities. Data from the Uppsala Conflict Data Program (UCDP).

G Twoway Influence between Verbal Conflict and ISIS Attacks

This section considers the possibility that the estimated effect of *Verbal Conflict* on *Attack Civilians* is an artifact of the reverse relationship, i.e., the tendency of governments to verbally respond to terror attacks and other acts of violent extremism (that is, *Attack Civilians* $\rightarrow$ *Verbal Conflict*). We note, first, that verbal government responses to terror attacks are the norm. Governments of all varieties routinely respond to terrorist attacks by issuing ultimatums, denunciations, threats, and other verbal attacks [33, 35, 36, 66, 67]. Further, government-extremist interactions are highly interdependent; each actor responds to the statements and actions of the other [49, 50, 68]. This interdependence means that we cannot plausibly isolate a specific exogenous verbal or material event and attempt to assess its independent causal impact. By definition, such events are not exogenous. A government's verbal response to a given terror attack may encourage that group to engage in more attacks, which in turn leads to further verbal conflict events, and so on. As noted by Enders and Sandler, "strategic interactions between terrorist and targeted governments, where actions are interdependent [...] cannot be analyzed as though one side were passive" [69, p. 13]. This interdependence means that we require an empirical model designed for endogenous systems.

In the main paper, we use CCF and Granger analysis as a first step to assess whether there is evidence that verbal conflict affects ISIS behavior. Both sets of models indeed show such evidence. However, as noted in Section B above, these models only measure correlations among lags and cannot determine causality or assess more complex relationships. For example, Table S2 shows that both the *Verbal Conflict* $\rightarrow$ *Attack Civilians* and *Attack Civilians* $\rightarrow$ *Verbal Conflict* relationships are highly statistically significant, with a larger F-statistic for the latter relationship than the former. However, neither the estimated significance of a modeled relationship nor the magnitude of the associated F-statistics can determine spuriousness in Granger models [31, 37, 38].

There are three approaches to analyzing dynamic systems that involve simultaneous interactions in both directions [32, 70, 71]. These are "(a) simultaneous equation models, (b) (vector) error correction models, and (c) vector autoregressions" [70, p. 76]. For the types of strategic interactions studied here, Bayesian structural VAR models are the best suited for capturing interactions in both directions [45, 72, 73, 74, 75, 76, 77]. They are particularly adept at treating parameter estimation and structural inference as a joint problem [78, pp. 140-170], and they offer "an unambiguous improvement over frequentist inference" [79].

The variables in an endogenous system often interact in complex ways. B-SVARs were "developed specifically to address the 'curse of dimensionality"' [80]. The ability of B-SVARs to model two-way causal relationships, forecast two-way outcomes, and enable graphical analysis of time-varying causal effects is a key methodological attraction [81, 82, 83, 84, 85]. Brandt et al. (2008) show that B-SVARs are appropriate for applications where we cannot plausibly assume that "no two variables in the model have contemporaneous, two-way causality within the period of analysis" [42]. Accordingly, B-SVARs have been widely applied to problems analogous to those encountered in the current study, such as political conflict and signaling between international actors [42], causality in political accountability [86], legislative politics and campaign contributions [39], mechanisms of influence between political institutions and the media [41], reciprocal relations between global powers [3], and dynamic relationships between corruption and the economy [87], as well as myriad applications in finance and economics [88, 89, 90]—and even natural phenomena like forecasting of precipitation events [91]. Across this research, scholars emphasize the utility of B-SVAR models in grappling with two-way causality. For example, in a study of how public opinion influences Hamas-

Israeli relations, Zeitzoff (2018) observes that "[r]ather than assuming, for instance, that shifts in public support only influence the conflict behavior of Israel and Hamas and not the reverse, a BSVAR allows for causality to flow both ways" [92]. In a study of economic sanctions and presidential approval, Webb (2017) implements a B-SVAR model because it "allows sanctions and presidential approval to instantaneously affect one another" [93]. In an extensive technical summary of B-SVAR models, with a particular focus on contemporaneous macro-level political and economic relationships, Brandt et al. (2009) emphasize that B-SVARs reflect the empirical reality that "politics is both a *cause and consequence* of economics" [94, emphasis in original].

Further, the specific procedure that we employ here—i.e., Gibbs sampling and impulse response functions, along with Granger causality as an initial step—has been established as particularly effective at capturing two-way causation beyond merely "allowing all variables to interact" [95, 96, 97]. The structural component, specifically, has been shown to allow information from theory and established literatures to be incorporated into the model, further enhancing the ability of SVARs to account for the interactions in both directions [30, 42, 71, 98].

Importantly, B-SVAR models include autoregressive lags of all variables in the system, which accounts, at least in part, for multi-day campaigns, follow-up attacks, and similar phenomena that might result in spurious correlations in the *Verbal Conflict* → *Attack Civilians* direction. For example, if ISIS attacks are part of a larger multi-day campaign, then we should observe temporal autocorrelation, where attacks one day predict attacks the following day. The B-SVAR model accounts for such dependencies with autoregressive lags. The estimated IRF for *Verbal Conflict* → *Attack Civilians* tells us the additional impact of an unexpected shock in *Verbal Conflict* on the *Attack Civilians* variable, where the systematic influence of other variables, including prior observations of *Attack Civilians* itself, are already modeled.

G.1 Additional empirical analysis

Much of the analyses in the main paper and this appendix contradict the possibility that the estimated *Verbal Conflict* → *Attack Civilians* relationship is a spurious artifact of the reverse effect. In Section C.5, for example, we consider structural identifications that incorporate government responses to ISIS attacks while excluding the effect of verbal conflict on ISIS attacks, and these specifications worsen rather than improve model fit. In Section F, we include a variable for multi-day ISIS campaigns, which controls for the possibility that government-initiated verbal attacks are merely responses to successive ISIS attacks, and the estimated IRFs remain essentially unchanged.

Here, we conduct additional analyses. First, we consider the possibility that the structural identifications, particularly for the Credibility model, somehow bias the estimate for the *Verbal Conflict* → *Attack Civilians* effect in favor of our hypothesis. The results for competing identifications, summarized in Section C.5, already show that this is not the case. To illustrate this point more clearly, in Figure S14 we plot the IRFs for *Verbal Conflict* → *Attack Civilians* across the Credibility, Government, and Government Plus structural identifications. The estimates are extremely similar across models. In fact, the estimated IRF in the Credibility model is slightly smaller than in the other models, contradicting the possibility of bias.

Second, we calculated the size of the effects in both directions of the *Verbal Conflict*/*Attack Civilians* relationship, which allows for a direct comparison of the two (Table S5). We find that a shock equivalent to 3.14 verbal attacks by governments results in an additional ISIS attack on civilians,

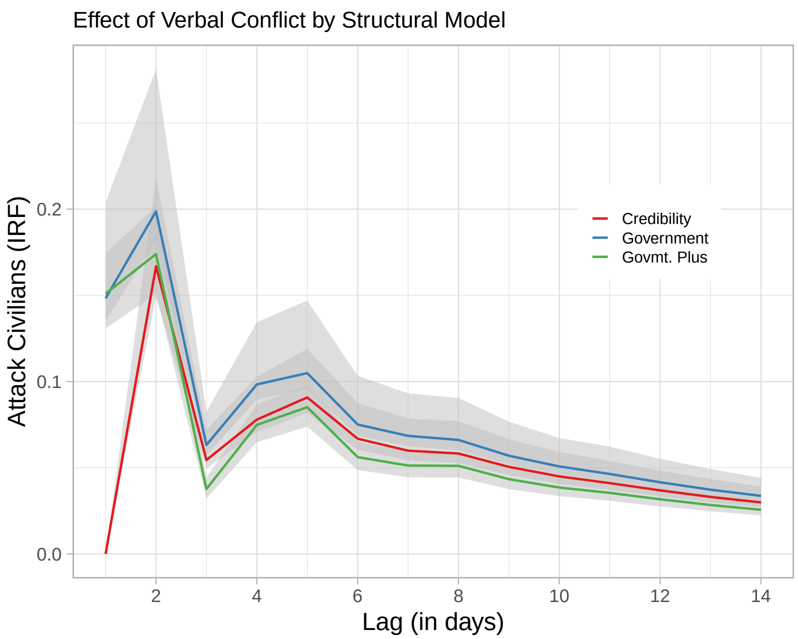

Figure S14: Impulse response functions for the B-SVAR model, comparing results from three structural identifications: Credibility, Government, and Government Plus.

while in the reverse direction, a shock equivalent to 9.30 attacks against civilians is needed to see one verbal attack in response by governments. Put differently, in terms of overall effect sizes, the causal relationship is in fact much stronger in the *Verbal Conflict* → *Attack Civilians* direction than in the reverse *Attack Civilians* → *Verbal Conflict* direction.

We also calculated effect sizes for both directions of the *Verbal Conflict*/*Attack Civilians* relationship within particular subsets of data. We argue in the main paper that, according to credibility-deficit logic, ISIS is most affected by verbal attacks when those attacks especially challenge its credibility, such as when attacks are initiated by major powers. Figure S15 illustrates IRFs in both directions for the main model and five subsets of data: (1) the 2014–2015 period; (2) verbal conflict that involves disapprovals; and (3) verbal conflict that involves threats; (4) verbal conflict initiated by members of the global anti-ISIS coalition; and (5) verbal conflict initiated by major powers.

In most cases, the IRFs suggest a relationship of similar intensity in both directions. However, the graphical representation does not allow direct comparison of the magnitude of the responses. It is standard practice in the literature to calculate effect sizes for the highest point in the IRFs, which are computed using 1-standard-deviation shocks [42, 71, 99]. The procedure is straightforward and involves converting the incoming shock from 1 standard deviation to a single event, and similarly calculating the maximum effect (highest point on the IRF) as a response. The purpose of this calculation is to enable more straightforward interpretation of "events" versus "standard deviation of events." Examining effect sizes reveals significant differences in magnitude. As Table S5 shows, for all five models the *Verbal Conflict* → *Attack Civilians* effect is substantially larger than the reverse effect—and, further, the magnitude of the effect of *Verbal Conflict*, relative to the reverse effect of *Attack Civilians*, is consistently larger in magnitude than in the main model.

If ISIS responses to verbal conflict are a spurious artifact of government reactions to ISIS attacks, then we should not observe systematic differences in the *Verbal Conflict*/*Attack Civilians* rela-

tionship within these subsets. We find, however, that in precisely those interactions where ISIS's credibility is most at stake, the effect of *Verbal Conflict* increases in magnitude relative to the reverse effect of *Attack Civilians*. If ISIS attacks on civilians are in fact unresponsive to governments' verbal actions, then we should not observe that those attacks are especially responsive to government statements that undermine the organization's credibility. Overall, these results contradict the possibility that the effect of verbal conflict is spurious to government reactions.

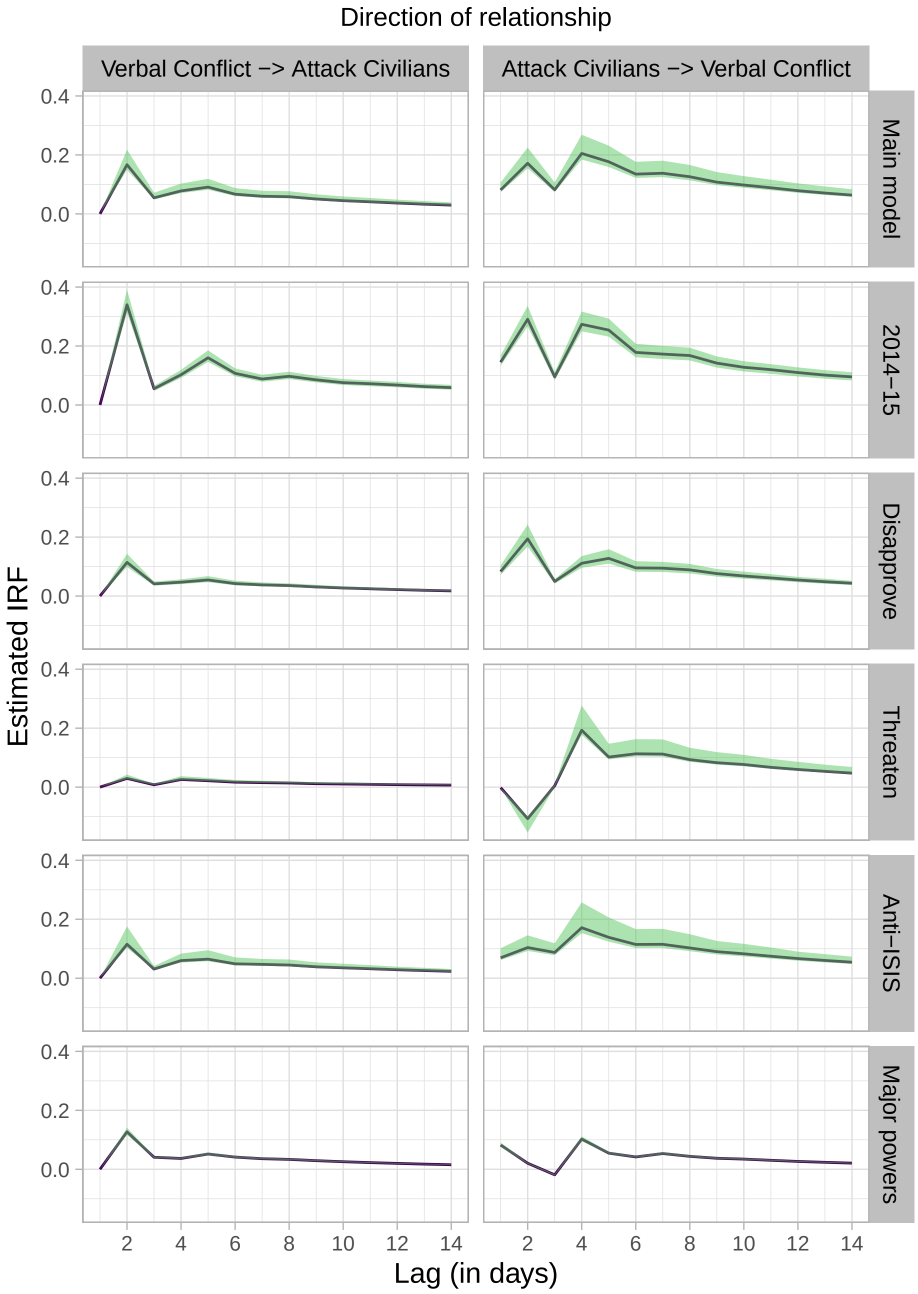

Figure S15: Two-way relationship between *Verbal Conflict* and *Attack Civilians* in specific types of interactions. Left column shows response in *Attack Civilians* to a shock in *Verbal Conflict*. Right column shows the response in *Verbal Conflict* to a shock in *Attack Civilians*. IRFs based on one-standard-deviation shocks.

Model	Verbal Conflict → Attack Civilians	Attack Civilians → Verbal Conflict	Ratio
Main model	3.14	9.30	2.96
Period 1 (2014-15)	1.53	6.59	4.29
Disapprove	3.41	13.22	3.88
Threaten	5.88	29.74	5.05
Anti-ISIS coalition	3.85	13.15	3.42
Major powers	2.65	29.03	10.95

Table S5: Peak observed effects of *Verbal Conflict* and *Attack Civilians* in both directions. Cells show number of *Verbal Conflict* events or *Attack Civilians* events associated with one additional event in the other variable. Lower values indicate effects of larger magnitude. The Ratio column illustrates how much larger the *Verbal Conflict* → *Attack Civilians* effect is than the reverse direction.

H Further Discussion

The main paper shows that ISIS responded violently to verbal attacks because it viewed such attacks as undermining its reputation and credibility. Here, we consider counterarguments and plausible alternative interpretations of our findings.

Why do leaders make threats in the first place if threats lead to humanitarian crises? Political scientists have consistently found that leaders make decisions primarily with an eye toward political survival [100]. Even when implementing foreign policy, leaders respond mainly to the interests and incentives generated by domestic "audiences," such as voting publics, interest groups, and legislatures [101, 102]. Leaders have an incentive to make bold, even provocative statements in the hopes of exhibiting strength and resolve, improving the audience's perception of their competence, and accruing political support [103]. Consequently, verbal attacks initiated by governments are likely motivated, at least in part, by domestic political interests.

At the same time, scholars of international relations have long emphasized the role of misperception in militarized crises [104]. Leaders cannot accurately assess the impact of their policies and statements on outside observers, and they often ignore crucial characteristics of the targets of signals [105]. Consequently, leaders may simply be unaware of the impact of their statements, or they may implicitly rely on motivated reasoning to dismiss those impacts [106]. Importantly, a government's underlying motivations for engaging in verbal attacks are analytically separate from the consequences of those attacks. Our main finding—that verbal conflict leads to attacks on civilians—is ultimately agnostic about why governments engage in such conflict.

Is the relationship between verbal conflict and civilian attacks due to governments making "preemptive" threats? If governments were aware of imminent ISIS attacks on civilian targets, and if they engaged in deterrent verbal threats in anticipation of those attacks, then our inferences regarding the effect of verbal conflict on civilians would essentially work in the opposite direction. However, this possibility is unlikely for two reasons. First, ISIS was highly capable in launching surprise attacks, often using a combination of technology, mobility, and local infrastructure to do so [107]. Second, if the estimated effect of verbal conflict on civilian attacks is in fact a result of governments making anticipatory threats, then we should observe a statistically significant relationship only between the "Threaten" root code and civilian attacks. However, we in fact find even stronger effects for the "Demand" and "Disapprove" root codes, neither of which is plausibly connected to this preemptive logic.

Is ISIS actually aware of the verbal attacks made against the organization? Can they access this information and respond to it? While we cannot directly measure the extent of the ISIS leadership's exposure to verbal attacks by foreign governments, all available information indicates that ISIS regularly monitored global news sources and social media activity [108], and the organization was highly sensitive to any commentary that cast doubt on its credibility. Between 2014 and 2016, ISIS's Ministry of Media published an English magazine, *Dabiq*, that incuded a section titledd "In the Words of the Enemy" that quoted statements on ISIS by foreign leaders, international organization, and policymakers. In addition to monthly publications, ISIS's control of multiple radio services, regional media bureaus throughout the Middle East and Africa, and an English-based production agency that translated Arabic newsletters, magazines, and advertisements demonstrated ISIS's strength in monitoring the statements and decisions of foreign governments and international actors. Scholarly and media sources alike regularly emphasized ISIS's "technological savvy" [109].

Did ISIS simply use civilian attacks as a way of exerting control over conquered territories? If ISIS' reliance on civilian attacks was primarily a means of controlling civilian populations, and not a response to verbal attacks by governments, then we should observe at least two outcomes. First, the number of civilian attacks should correspond to the amount of territory controlled by ISIS. In fact, we observe that although the extent of ISIS' territorial control was greatest in mid-2015, attacks on civilians experienced a lull during this period. Notably, verbal conflict was also low during this same period. Second, we should observe that the estimated relationship between *Verbal Conflict* and *Attack Civilians* is statistically insignificant, or at least constant over time; that is, civilian attacks should be a function of territorial control, not verbal attacks initiated by governments. In fact, we find that the relationship between *Verbal Conflict* and *Attack Civilians* is highly significant, and the magnitude of this relationship is greatest in the early stages of the conflict, when ISIS did not yet control large amounts of territory but was feverishly attempting to establish its reputation for brutality.

Further, if the significant estimated relationship between *Verbal Conflict* and *Attack Civilians* were somehow epiphenomenal to ISIS's efforts at controlling territory, then we should observe that that relationship is strongest in those areas where ISIS was most active at establishing or maintaining control over conquered local populations. Yet, as illustrated by Figure S11(c) above, the opposite is true.

Finally, some contextual evidence suggests citizens living in conquered territories viewed ISIS as a legitimate government due to its ability to restore security and provide basic services to civilians, not through fear that stemmed from civilian attacks. Through its media publications, ISIS proclaimed its responsibility for caring for its citizens. ISIS police and security implemented rule of law and punished criminals [110]. ISIS managed to provide electricity, clean streets, and free health care services to civilians in ways that the governments of Iraq and Syria failed [111]. These benefits allowed some "citizens" of the Islamic State to feel as if their lives were better under ISIS, which may have legitimized ISIS control over conquered territories [112, 113, 114].

Was ISIS "just being ISIS," i.e., behaving erratically and attacking targets randomly due to their extreme ideology? Such behavior would not be strategic nor would it make sense given our knowledge of extremist organizations or ISIS in particular [50, 115]. It would not be effective under any theoretical model. Furthermore, our findings depict *responses to* government actions throughout the period of interest. If ISIS was not responsive to government actions and instead simply acted randomly, we would not detect any such responses. That is, our estimates would be statistically indistinguishable from zero.

Did ISIS use civilian attacks to discredit governments who engaged in verbal conflict, specifically by targeting the civilians, officials, or personnel of those governments? While our analysis includes all civilian attacks perpetrated by ISIS, the vast majority of these attacks occurred in Iraq, Syria, and other regions where ISIS exercised some degree of territorial control, and the vast majority of the civilians harmed by ISIS were residents of these areas. By contrast, the vast majority of verbal attacks came from western governments, such as the United States, France, and the United Kingdom. Despite high-profile incidents, such as the beheading of US journalist James Foley, ISIS had relatively few opportunities to directly target civilians from those countries primarily engaged in verbal conflict.

Didn't ISIS benefit from verbal conflict—especially conflict initiated by powerful governments—by leveraging that attention to increase its public profile and attract recruits? As discussed in the main paper, at least some instances of verbal conflict were used

by ISIS as recruitment tools. Regardless of their motivations, when government leaders directed verbal conflict toward ISIS, they may have inadvertently given the organization fuel for its global propaganda campaign [109]. However, this possibility does not undermine our argument or empirical findings. First, the possibility that ISIS repurposed verbal attacks as propaganda is not inconsistent with the logic of a credibility deficit. That is, although attention from governments may have raised ISIS's profile, the content of the statements made by those governments was also capable of undermining the organization's sense of credibility.

Second, and relatedly, ISIS responded to different types of verbal conflict in different ways. As illustrated by the examples from *Dabiq*, ISIS particularly favored propagandizing verbal statements that portrayed the organization as unexpectedly fearsome or intimidating. When ISIS chose to publicize government statements that condemned, threatened, or otherwise challenged the organization, it nearly always paired those statements with descriptions of follow-up actions that the organization took in response to the offending statement, such as an execution, abduction, or armed attack. In short, irrespective of whether verbal attacks were eventually utilized as propaganda, they also were often viewed by the organization as a challenge to its credibility that warranted a response.

Finally, if verbal attacks were considered by ISIS to be singularly beneficial to the organization, with no negative impacts on its credibility, then ISIS would have no strategic incentive to respond to those attacks by attacking civilians. That is, we should observe no significant empirical correlation between *Verbal Conflict* and *Attack Civilians*. Thus, the possibility that ISIS only benefited from verbal conflict is inconsistent with the results of the empirical analysis.

References

[1] Sean P. O'Brien. Crisis early warning and decision support: Contemporary approaches and thoughts on future research. *International Studies Review*, 12(1):87–104, 2010.

[2] Patrick T Brandt, Vito D'Orazio, Latifur Khan, Yi-Fan Li, Javier Osorio, and Marcus Sianan. Conflict forecasting with event data and spatio-temporal graph convolutional networks. *International Interactions*, 48(4):800–822, 2022.

[3] Joshua S Goldstein and John R Freeman. Us-soviet-chinese relations: Routine, reciprocity, or rational expectations?. *American Political Science Review*, 85(01):17–35, 1991.

[4] Peter Tikuisis, David Carment, and Yiagadeesen Samy. Prediction of Intrastate Conflict Using State Structural Factors and Events Data. *Journal of Conflict Resolution*, 57(3):410–444, 2013.

[5] Nils W. Metternich, Cassy Dorff, Max Gallop, Simon Weschle, and Michael D. Ward. Antigovernment Networks in Civil Conflicts: How Network Structures Affect Conflictual Behavior. *American Journal of Political Science*, 57(4):892–911, 2013.

[6] Michael D. Ward and Andreas Beger. Lessons from near real-time forecasting of irregular leadership changes. *Journal of Peace Research*, 54(2):141–156, 2017.

[7] Nils W. Metternich, Shahryar Minhas, and Michael D. Ward. Firewall? or Wall on Fire? A Unified Framework of Conflict Contagion and the Role of Ethnic Exclusion. *Journal of Conflict Resolution*, 61(6):1151–1173, 2017.

[8] Shahryar Minhas, Peter D Hoff, and Michael D Ward. A new approach to analyzing coevolving longitudinal networks in international relations. *Journal of Peace Research*, 53(3):491–505, 2016.

[9] Morgan R. Frank, Nick Obradovich, Lijun Sun, Wei Lee Woon, Brad L. LeVeck, and Iyad Rahwan. Detecting reciprocity at a global scale. *Science Advances*, 4(1):eeaao5348, 2018.

[10] Wei Wang, Ryan Kennedy, David Lazer, and Naren Ramakrishnan. Growing Pains for Global Monitoring of Societal Events: Automated event coding raises promise and concerns. *Science*, 353(6307):1502–1504, 2016.

[11] Vito D'orazio and James E. Yonamine. Kickoff to conflict: A sequence analysis of intra-state conflict-preceding event structures. *PLoS ONE*, 10(5):1–21, 2015.

[12] Clionadh Raleigh, rew Linke, Håvard Hegre, and Joakim Karlsen. Introducing acled: An armed conflict location and event dataset. *Journal of peace research*, 47(5):651–660, 2010.

[13] Ralph Sundberg, Kristine Eck, and Joakim Kreutz. Introducing the ucdp non-state conflict dataset. *Journal of peace research*, 49(2):351–362, 2012.

[14] Gary LaFree and Laura Dugan. Introducing the global terrorism database. *Terrorism and political violence*, 19(2):181–204, 2007.

[15] Justin Grimmer and Brandon M. Stewart. Text as data: The promise and pitfalls of automatic content analysis methods for political texts. *Political Analysis*, 21(3):267–297, 2013.

[16] Michael D Ward, Andreas Beger, Josh Cutler, Matt Dickenson, Cassy Dorff, and Ben Radford. Comparing GDELT and ICEWS event data. 2013.

[17] N. Johnson, A. Hitchman, D. Phan, and L. Smith. Self-exciting point process models for political conflict forecasting. *European Journal of Applied Mathematics*, 29(4):685–707, 2018.

[18] Janet E. Wedgwood, Alicia Ruvinsky, and Timothy Siedlecki. What Lies Beneath. In Denise M. Nicholson and Dylan D. Schmorrow, editors, *Design for Cross-Cultural Activities, Part II*, pages 64–73. CRC Press, London, 2013.

[19] Nils B. Weidmann. On the Accuracy of Media-based Conflict Event Data. *Journal of Conflict Resolution*, 59(6):1129–1149, 2015.

[20] Benjamin E. Bagozzi, Patrick T. Brandt, John R. Freeman, Jennifer S. Holmes, Alisha Kim, Agustin Palao Mendizabal, and Carly Potz-Nielsen. The Prevalence and Severity of Underreporting Bias in Machine- and Human-Coded Data. *Political Science Research and Methods*, 7(3):641–649, 2019.

[21] Michael D Ward. Can We Predict Politics? Toward What End? *Journal of Global Security Studies*, 1(1):80–91, 2016.

[22] Michael D Ward, Nils W Metternich, Cassy L Dorff, Max Gallop, Florian M Hollenbach, Anna Schultz, and Simon Weschle. Learning from the past and stepping into the future: Toward a new generation of conflict prediction. *International Studies Review*, 15(4):473–490, 2013.

[23] Philip A Schrodt. *CAMEO: Conflict and Mediation Event Observations Event and Actor Codebook*. Event Data Project, Department of Political Science, Pennsylvania State University, 1.1b3 edition, mar 2012.

[24] Deborah J Gerner, Philip A Schrodt, Omür Yilmaz, and Rajaa Abu-Jabr. Conflict and mediation event observations (cameo): A new event data framework for the analysis of foreign policy interactions. *Annual Meeting of the International Studies Association, New Orleans*, 2002.

[25] Philip A. Schrodt. text_to_CAMEO. Available at `https://github.com/openeventdata/text_to_CAMEO`, 2021. Python 3.5, 8 February, 2021.

[26] R.H. Shumway and D.S. Stoffer. *Time Series Analysis and Its Applications: With R Examples*. Springer Verlag, 2010.

[27] Iliyan R Iliev and Patrick T Brandt. Money and rhetoric: Energy sector dynamics in us senate committee. *The Social Science Journal*, Feb:1–18, 2020.

[28] Gonzalo Caballero and Marcos Álvarez-Díaz. The procyclicality of political trust in spain. *Panoeconomicus*, 65(1):21–36, 2018.

[29] Chung-hong Chan and King-Wa Fu. Predicting political polarization from cyberbalkanization: Time series analysis of facebook pages and opinion poll during the hong kong occupy movement. In *Proceedings of the ACM Web Science Conference*, pages 1–2, 2015.

[30] Patrick T. Brandt and John T. Williams. *Multiple time series models*, volume 148. Sage Publications, Incorporated, 2006.

[31] John R Freeman. Granger causality and the times series analysis of political relationships. *American Journal of Political Science*, 27(2):327–358, 1983.

[32] John R Freeman, John T Williams, and Tse-min Lin. Vector autoregression and the study of politics. *American Journal of Political Science*, 33(4):842–877, 1989.

[33] Helen Norton. Government speech and the war on terror. *Fordham L. Rev.*, 86:543, 2017.

[34] Scott E Atkinson, Todd Sandler, and John Tschirhart. Terrorism in a bargaining framework. *the Journal of Law and Economics*, 30(1):1–21, 1987.

[35] Thomas Jensen. National responses to transnational terrorism: Intelligence and counterterrorism provision. *Journal of Conflict Resolution*, 60(3):530–554, 2016.

[36] Joseph M Brown. Force of words: the role of threats in terrorism. *Terrorism and political violence*, 32(7):1527–1549, 2020.

[37] Zonglu He and Koichi Maekawa. On spurious granger causality. *Economics Letters*, 73(3):307–313, 2001.

[38] MV Hood, Quentin Kidd, and Irwin L Morris. Two sides of the same coin? employing granger causality tests in a time series cross-section framework. *Political Analysis*, 16(3):324–344, 2008.

[39] Iliyan R Iliev. The power dynamics of campaign contributions and legislative rhetoric. *Interest Groups & Advocacy*, July, 2021.

[40] Thomas Sattler, John R Freeman, and Patrick T Brandt. Political accountability and the room to maneuver: A search for a causal chain. *Comparative political studies*, 41(9):1212–1239, 2008.

[41] George C Edwards and B Dan Wood. Who influences whom? the president, congress, and the media. *American Political Science Review*, 93(02):327–344, 1999.

[42] Patrick T. Brandt, Michael Colaresi, and John R. Freeman. The dynamics of reciprocity, accountability, and credibility. *Journal of Conflict Resolution*, 52(3):343–374, 2008.

[43] Thomas F Cooley and Stephen F LeRoy. Atheoretical macroeconometrics: a critique. *Journal of Monetary Economics*, 16(3):283–308, 1985.

[44] Patrick T. Brandt and John R. Freeman. Advances in bayesian time series modeling and the study of politics: Theory testing, forecasting, and policy analysis. *Political Analysis*, 14(1):1–36, 2006.

[45] Christopher A Sims and Tao Zha. Bayesian methods for dynamic multivariate models. *International Economic Review*, 39(4):949–968, 1998.

[46] Patrick Brandt and W Ryan Davis. Package 'msbvar'. *Relatório técnico, Comprehensive R Archive*, 2015.

[47] Paul K Huth. Deterrence and international conflict: Empirical findings and theoretical debates. *Annual Review of Political Science*, 2(1):25–48, 1999.

[48] James D Fearon. Rationalist Explanations for War. *International Organization*, 49(3):379–414, 1995.

[49] Harvey E. Lapan and Todd Sandler. Terrorism and signalling. *European Journal of Political Economy*, 9(3):383–397, 1993.

[50] Andrew H Kydd and Barbara F Walter. The Strategies of Terrorism. *International Security*, 31(1):49–80, 2006.

[51] Joshua S Goldstein, Jon C Pevehouse, Deborah J Gerner, and Shibley Telhami. Reciprocity, triangularity, and cooperation in the middle east, 1979-97. *Journal of Conflict Resolution*, 45(5):594–620, 2001.

[52] Joshua D Kertzer and Brian C Rathbun. Fair is fair: Social preferences and reciprocity in international politics. *World Politics*, 67(4):613–655, 2015.

[53] Joshua S Goldstein and Jon C Pevehouse. Reciprocity, bullying, and international cooperation: Time-series analysis of the bosnia conflict. *American Political Science Review*, 91(3):515–529, 1997.

[54] Sheen Rajmaira and Michael D Ward. Evolving foreign policy norms: Reciprocity in the superpower triad. *International Studies Quarterly*, 34(4):457–475, 1990.

[55] Robert E Kass and Adrian E Raftery. Bayes factors. *Journal of the american statistical association*, 90(430):773–795, 1995.

[56] Christopher A Sims and Tao Zha. Error bands for impulse responses. *Econometrica*, 67(5):1113–1155, 1999.

[57] Christopher A Sims, Daniel F Waggoner, and Tao Zha. Methods for inference in large multiple-equation markov-switching models. *Journal of Econometrics*, 146(2):255–274, 2008.

[58] Christopher A Sims. Are forecasting models usable for policy analysis? *Federal Reserve Bank of Minneapolis Quarterly Review*, 10(1):2–16, 1986.

[59] Daniel F Waggoner and Tao Zha. A gibbs sampler for structural vector autoregressions. *Journal of Economic Dynamics and Control*, 28(2):349–366, 2003.

[60] Daniel F Waggoner and Tao Zha. Likelihood preserving normalization in multiple equation models. *Journal of Econometrics*, 114(2):329–347, 2003.

[61] Adrian E Raftery and Steven Lewis. How many iterations in the gibbs sampler? Technical report, WASHINGTON UNIV SEATTLE DEPT OF STATISTICS, 1991.

[62] Philip Heidelberger and Peter D Welch. Simulation run length control in the presence of an initial transient. *Operations Research*, 31(6):1109–1144, 1983.

[63] Stephen P Brooks and Andrew Gelman. General methods for monitoring convergence of iterative simulations. *Journal of computational and graphical statistics*, 7(4):434–455, 1998.

[64] Andrew Gelman and Donald B Rubin. Inference from iterative simulation using multiple sequences. *Statistical science*, 7(4):457–472, 1992.

[65] Samuel P Huntington. *The clash of civilizations?* Simon & Schuster, New York, 1996.

[66] Laura Dugan and Erica Chenoweth. Government actions in terror environments (gate): A methodology that reveals how governments behave toward terrorists and their constituencies. In V.S. Subrahmanian, editor, *Handbook of computational approaches to counterterrorism*, pages 465–486. Springer, 2012.

[67] Ronald D Crelinsten. Analysing terrorism and counter-terrorism: A communication model. *Terrorism and political violence*, 14(2):77–122, 2002.

[68] Navin A. Bapat. State Bargaining with Transnational Terrorist Groups. *International Studies Quarterly*, 50(1):213–230, 2006.

[69] Walter Enders and Todd Sandler. *The political economy of terrorism.* Cambridge University Press, New York, 2011.

[70] Thomas Sattler, Patrick T Brandt, John R Freeman, et al. Democratic accountability in open economies. *Quarterly Journal of Political Science*, 5(1):71–97, 2010.

[71] Patrick T Brandt and John T Williams. *Multiple time series models.* Sage, 2007.

[72] David O Cushman and Tao Zha. Identifying monetary policy in a small open economy under flexible exchange rates. *Journal of Monetary economics*, 39(3):433–448, 1997.

[73] Eric M Leeper, Christopher A Sims, Tao Zha, Robert E Hall, and Ben S Bernanke. What does monetary policy do? *Brookings papers on economic activity*, 1996(2):1–78, 1996.

[74] David W Sims, Emily J Southall, Nicolas E Humphries, Graeme C Hays, Corey JA Bradshaw, Jonathan W Pitchford, Alex James, Mohammed Z Ahmed, Andrew S Brierley, Mark A Hindell, et al. Scaling laws of marine predator search behaviour. *Nature*, 451(7182):1098–1102, 2008.

[75] Markku Lanne and Jani Luoto. Data-driven inference on sign restrictions in bayesian structural vector autoregression. 2016.

[76] Maria Kalli and Jim E Griffin. Bayesian nonparametric vector autoregressive models. *Journal of econometrics*, 203(2):267–282, 2018.

[77] Helmut Lütkepohl and Tomasz Woźniak. Bayesian inference for structural vector autoregressions identified by markov-switching heteroskedasticity. *Journal of Economic Dynamics and Control*, 113:103862, 2020.

[78] Lutz Kilian and Helmut Lütkepohl. *Structural vector autoregressive analysis.* Cambridge University Press, 2017.

[79] Christiane Baumeister and James D Hamilton. Sign restrictions, structural vector autoregressions, and useful prior information. *Econometrica*, 83(5):1963–1999, 2015.

[80] Elena Deryugina and Alexey A Ponomarenko. A large bayesian vector autoregression model for russia. 2014.

[81] Warsono Warsono, Dian Kurniasari, and Mustofa Usman. Analysis of dynamic structure, granger causality and forecasting with vector autoregression (var) models on credit risk data. *Science International Lahore*, 30(1):7–16, 2018.

[82] Salma Keshtkaran and Farzane Bagheri. The relationship between government size and economic growth in iran; bivariate and trivariate causality testing. *Journal of Economics and Behavioral Studies*, 4(5):268–276, 2012.

[83] Luca Agnello, Vitor Castro, Fredj Jawadi, and Ricardo M Sousa. How does monetary policy respond to the dynamics of the shadow banking sector? *International Journal of Finance & Economics*, 25(2):228–247, 2020.

[84] Vijaykumar Dhannur and Ashwin R John. Foreign direct investment and export performance using the hierarchical bayesian vector autoregression framework. *Managerial and Decision Economics*, 42(7):1679–1685, 2021.

[85] Libo Yin and Xiyuan Ma. Causality between oil shocks and exchange rate: a bayesian, graph-based var approach. *Physica A: Statistical Mechanics and its Applications*, 508:434–453, 2018.

[86] Thomas Sattler, John R Freeman, and Patrick T Brandt. Political accountability and the room to maneuver: A search for a causal chain. *Comparative Political Studies*, 41(9):1212–1239, 2008.

[87] Seifallah Sassi and Amira Gasmi. The dynamic relationship between corruption—inflation: Evidence from panel vector autoregression. *The Japanese Economic Review*, 68:458–469, 2017.

[88] Lutz Kilian. Structural vector autoregressions. In *Handbook of research methods and applications in empirical macroeconomics*, pages 515–554. Edward Elgar Publishing, 2013.

[89] Chaido Dritsaki and Melina Dritsaki-Bargiota. The causal relationship between stock, credit market and economic development: an empirical evidence for greece. *Economic Change and Restructuring*, 38:113–127, 2005.

[90] Giorgio E Primiceri. Time varying structural vector autoregressions and monetary policy. *The Review of Economic Studies*, 72(3):821–852, 2005.

[91] Kaixun Hua and Dan A Simovici. Long-lead term precipitation forecasting by hierarchical clustering-based bayesian structural vector autoregression. In *2016 IEEE 13th International Conference on Networking, Sensing, and Control (ICNSC)*, pages 1–6. IEEE, 2016.

[92] Thomas Zeitzoff. Does social media influence conflict? evidence from the 2012 gaza conflict. *Journal of Conflict Resolution*, 62(1):29–63, 2018.

[93] Clayton Webb. Power politics or public pandering? an empirical investigation of economic sanctions and presidential approval. *International Interactions*, 44(3):491–509, 2018.

[94] Patrick T Brandt and John R Freeman. Modeling macro-political dynamics. *Political Analysis*, 17(2):113–142, 2009.

[95] Liana Jacobi, Dan Zhu, and Mark Joshi. Estimating posterior sensitivities with application to structural analysis of bayesian vector autoregressions. *Journal of Business & Economic Statistics*, (just-accepted):1–47, 2024.

[96] Ali Rezazadeh, Shahab Jahangiri, and Fahmideh Fattahi. The impact of financial inclusion shocks on financial cycles with emphasis on financial stability: A panel-var approach. *Iranian Economic Review*, 27(3):1007–1032, 2023.

[97] Kyriaki-Argyro Tsioptsia, Eleni Zafeiriou, Dimitrios Niklis, Nikolaos Sariannidis, and Constantin Zopounidis. The corporate economic performance of environmentally eligible firms nexus climate change: An empirical research in a bayesian var framework. *Energies*, 15(19):7266, 2022.

[98] Lam Nguyen. Bayesian inference in structural vector autoregression with sign restrictions and external instruments. *Available at SSRN 4680099*, 2022.

[99] Patrick T Brandt and Todd Sandler. What do transnational terrorists target? has it changed? are we safer? *Journal of Conflict Resolution*, 54(2):214–236, 2010.

[100] Bruce de Mesquita, Alastair Smith, Randolph M Siverson, and James D Morrow. *The Logic of Political Survival.* The MIT Press, Cambridge, MA, 2003.

[101] James D Fearon. Domestic Political Audiences and the Escalation of International Disputes. *American Political Science Review*, 88(3):577–592, 1994.

[102] Robert D Putnam. Diplomacy and Domestic Politics: The Logic of 2-Level Games. *International Organization*, 42(3):427–460, 1988.

[103] Alastair Smith. International crises and domestic politics. *The American Political Science Review*, 92(3):623–638, 1998.

[104] Robert Jervis. *Perception and misperception in international politics.* Princeton University Press, Princeton, NJ, 1976.

[105] Keren Yarhi-Milo, Joshua D. Kertzer, and Jonathan Renshon. Tying Hands, Sinking Costs, and Leader Attributes. *Journal of Conflict Resolution*, 62(10):2150–2179, 2018.

[106] Peter Beattie and Danielle Snider. Knowledge in international relations: Susceptibilities to motivated reasoning among experts and non-experts. *Journal of Social and Political Psychology*, 7(1):172–191, Mar. 2019.

[107] Andrea Beccaro. Modern irregular warfare: The isis case study. *Small Wars & Insurgencies*, 29(2):207–228, 2018.

[108] James P. Farwell. The media strategy of isis. *Survival*, 56(6):49–55, 2014.

[109] Asma Shakir Khawaja and Asma Hussain Khan. Media strategy of isis. *Strategic Studies*, 36(2):104–121, 2016.

[110] Mara Revkin and William McCants. Experts weigh in: Is isis good at governing? *The Brookings Institution*, 2015.

[111] Mara R Revkin. When terrorists govern: Protecting civilians in conflicts with state-building armed groups. *Harv. Nat'l Sec. J.*, 9:100, 2018.

[112] Karl Kaltenthaler, Daniel Silverman, and Munqith Dagher. Identity, ideology, and information: The sources of iraqi public support for the islamic state. *Studies in Conflict & Terrorism*, 41(10):801–824, 2018.

[113] Daniel Byman. Understanding the Islamic State—A Review Essay. *International Security*, 40(4):127–165, 2016.

[114] Aymenn Al-Tamimi. The evolution in islamic state administration: The documentary evidence. *Perspectives on Terrorism*, 9(4):117–129, 2015.

[115] Todd Sandler. The analytical study of terrorism: Taking stock. *Journal of Peace Research*, 51(2):257–271, 2014.
